# Modified SureSelect^QXT^ Target Enrichment Protocol for Illumina Multiplexed Sequencing of FFPE Samples

**DOI:** 10.1186/s12575-018-0084-7

**Published:** 2018-10-12

**Authors:** J. M. Rosa-Rosa, T Caniego-Casas, S Leskela, G Muñoz, F del Castillo, P Garrido, J Palacios

**Affiliations:** 1 CIBER-ONC, Instituto de Salud Carlos III, Madrid, Spain; 2 Instituto Ramón y Cajal de Investigación Sanitaria, Madrid, Spain; 3 Servicio de Genética, Hospital Universitario Ramón y Cajal, Madrid, Spain; 4 CIBER-ER, Instituto de Salud Carlos III, Madrid, Spain; 5 Medical Oncology Department, Hospital Universitario Ramón y Cajal, Madrid, Spain; 6 Facultad de Medicina, Universidad de Alcalá de Henares, Madrid, Spain; 7 Servicio de Anatomía Patológica, Hospital Ramón y Cajal, Ctra. Colmenar Viejo km 9,100, 28034 Madrid, Spain

**Keywords:** NGS, FFPE samples, SureSelect^QXT^, Optimization, Protocol

## Abstract

**Background:**

Personalised medicine is nowadays a major objective in oncology. Molecular characterization of tumours through NGS offers the possibility to find possible therapeutic targets in a time- and cost-effective way. However, the low quality and complexity of FFPE DNA samples bring a series of disadvantages for massive parallel sequencing techniques compared to high-quality DNA samples (from blood cells, cell cultures, etc.).

**Results:**

We performed several experiments to understand the behaviour of FFPE DNA samples during the construction of SureSelect^QXT^ libraries. First, we designed a quality checkpoint for FFPE DNA samples based on the quantification of their amplification capability (qcPCR). We observed that FFPE DNA samples can be classified according to DIN value and qcPCR concentration into unusable, or low-quality (LQ) and good-quality (GQ) DNA. For GQ samples, we increased the amount of input DNA to 150 ng and the digestion time to 30 min, whereas for LQ samples, we used 50 ng of DNA as input but we decreased the digestion time to 1 min. In all cases, we increased the cycles of the pre-hyb PCR to 10 but decreased the cycles of the post-hyb PCR to 8. In addition, we confirmed that using half of the volume of reagents can be beneficial. Finally, in order to obtain better results, we designed a decision flow-chart to achieve a seeding concentration of 12–14 pM for MiSeq Reagent Kit v2.

**Conclusions:**

Our experiments allowed us to unveil the behaviour of low-quality FFPE DNA samples during the construction of SureSelect^QXT^ libraries. Sequencing results showed that, using our modified SureSelect^QXT^ protocol, the final percentage of usable reads for low-quality samples was increased more than three times allowing to reach median depth/million reads values of 76.35. This value is equivalent to ~ 0.9 and ~ 0.7 of the values obtained for good-quality FFPE and high-quality DNA respectively.

**Electronic supplementary material:**

The online version of this article (10.1186/s12575-018-0084-7) contains supplementary material, which is available to authorized users.

## Background

Personalized precision therapy has become the aim of nowadays medicine. The molecular characterization of malignant tumours is now an extended practice to obtain information about prognosis and therapeutic targets [1, 2]. In addition, with the recent development of massive parallel sequencing (or next generation sequencing, NGS), genetic characterization of tumours is now more time- and cost-efficient as it never was before.

There are innumerable scientific articles based on the enrichment of target regions and NGS. Different techniques and protocols have arisen to offer a wide variety of solution, and among these solutions, capture-based enrichment protocols have a predominant role in this scenario, as can be seen in publications from the TCGA consortium [3–8]. Nonetheless, hybridization time necessary to perform standard protocols (between 16 and 24 h) has slowed down their application in exigent diagnostic fields such as oncology. Therefore, faster lab-techniques have been developed and offered by commercial manufacturers, such as multiplex-PCR based or restriction-enzymes based solutions [9, 10].

DNA extracted from formalin-fixed paraffin-embedded (FFPE) samples is always fragmented (ranging from < 100 bp to > 3 kb), and contains cross-links due to chemical modifications [11]. Although some strong recommendations about the treatment of tissues during fixation and embedment (FFPE process) have recently been reported [12–14], most of the available specimens have been treated in a different, less optimal, way. Subsequently, it may be difficult to achieve the desired results in all key parameters under consideration by using these alternative lab techniques, as previously reported [14–16]. It is important to highlight that using NGS-optimised DNA/RNA extraction protocols might improve final results [17, 18].

In this research, we tested a novel capture-based technique (SureSelect^QXT^) from Agilent technologies. SureSelect^QXT^ offers a faster library preparation protocol and avoids the necessity of DNA fragmentation-by-sonication due to the use of modified transposase enzymes. However, since it is not possible to change the characteristics of our available FFPE samples, we decided to modify the protocol in order to optimise the results.

## Results and Discussion

Here we include and describe the results obtained from the experiments that composed the different modules detailed in Methods:

### Library Preparation

**Fig. 1:**
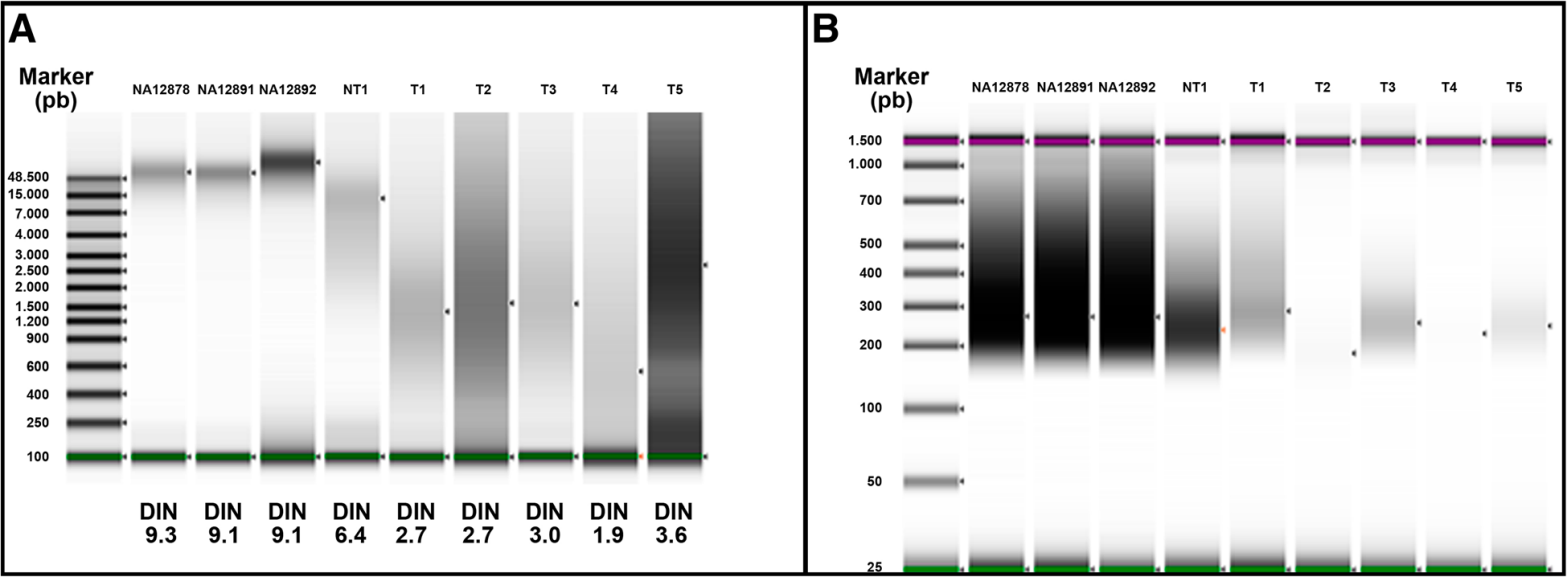
Quality and integrity of the 9 DNA samples with the Agilent 2200 Tape Station and Genomic DNA Screen Tape. **a** Genomic DNA samples. **b** Digested, amplified and purified DNA samples

**Fig. 2:**
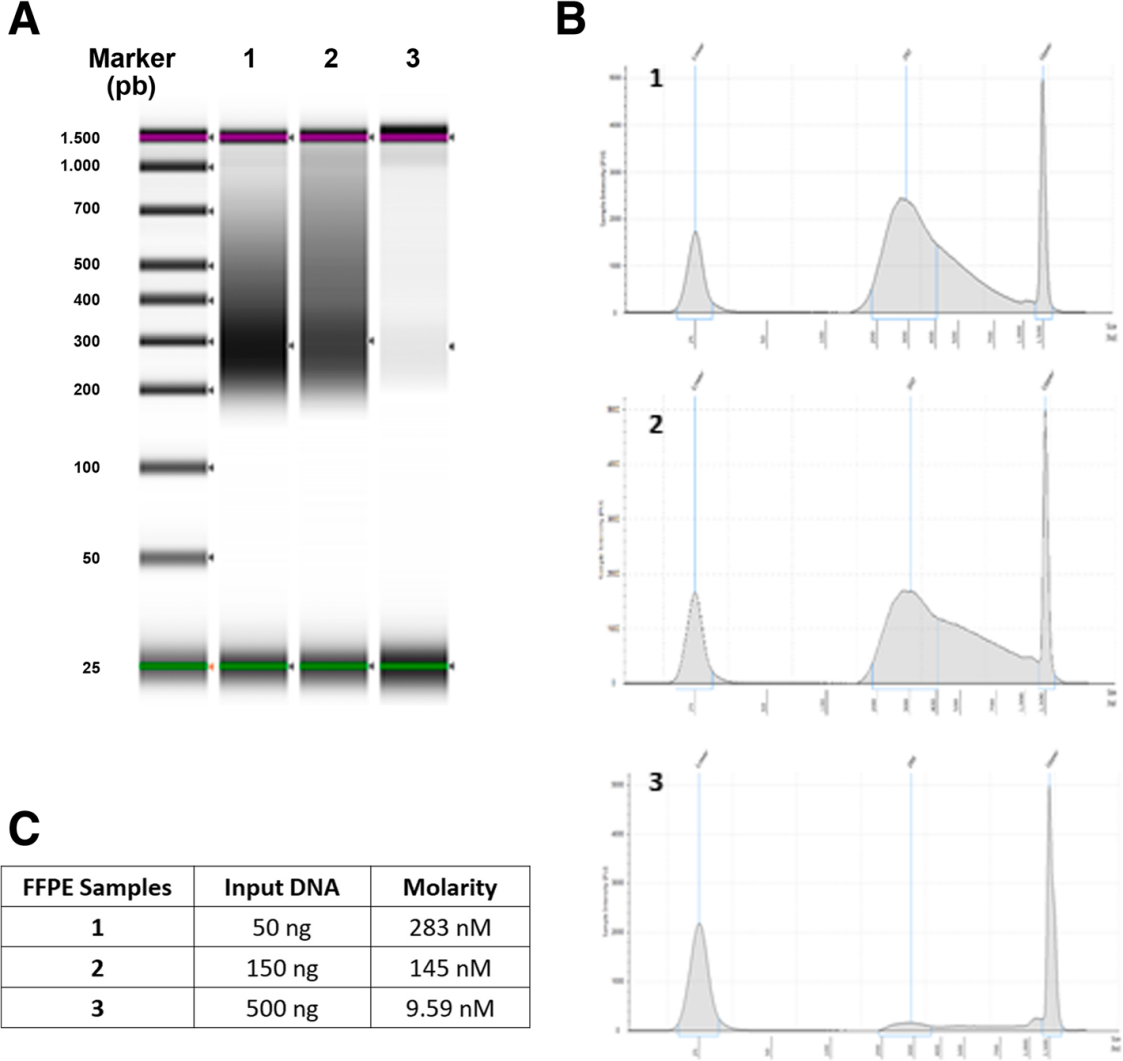
Quantification and qualification of library DNA with the Agilent 2200 Tape station and D1000 Screen Tape using 50 (1), 150 (2) and 500 (3) ng of DNA input. **a** Gel image. **b** Electropherogram. **c** Table with library concentration estimations

**Fig. 3:**
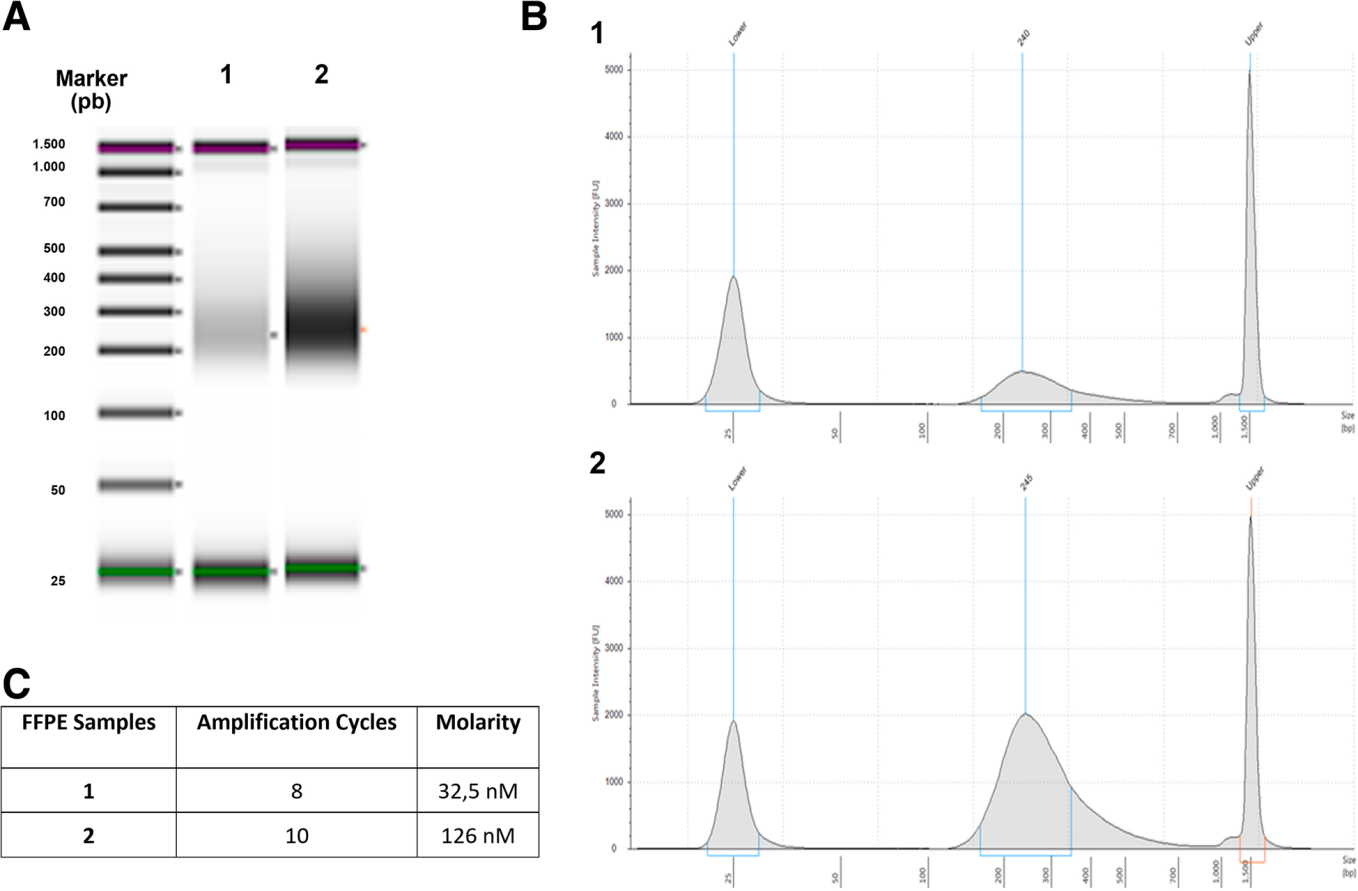
Quantification and qualification of library DNA with the Agilent 2200 Tape station and D1000 Screen Tape using 8 (1) or 10 (2) cycles during pre-hybridization PCR. **a** Gel image. **b** Electropherogram. **c** Table with library concentration estimations

In order to assess DNA integrity, we measured the DNA integrity number (DIN) for each sample (Fig. 1a). Results showed that the quality of the DNA extracted from FFPE samples was low, ranging from 1.9 to 6.4. However, we still performed the first steps of the commercial SureSelect^QXT^ protocol in order to obtain the size profiles of the digested and PCR-amplified fragments (Fig. 1b). Since the level of DNA degradation seems to be critical, our first approach was to increase the quantity of genomic DNA as input for “Fragment and adaptor-tag the genomic DNA samples” step. This can help in achieving a greater number of fragments with a suitable size. We used inputs of 50, 150 and 500 ng for sample NT1, because this FFPE sample presented the best DIN value. After the “Amplify the adaptor-tagged DNA library” step, we assessed quantity and quality of the libraries (Fig. 2) and we observed that higher input of DNA led to lower library concentration. Thus, our next aim was to obtain sequenceable libraries for those samples with lower DIN values. We observed that a higher amount of library prior to the hybridization step was needed. Thus we increased up to 10 the cycles at the pre-hyb PCR program keeping standard library preparation conditions (50 ng of input DNA). We used sample T1, which presented a low DIN and was previously sequenced using the Haloplex technique from Agilent [1]. Results showed a noticeable increment in the concentration of the library when using 10 cycles compared to the standard 8 cycles (Fig. 3) with 50 ng of input DNA. Libraries were constructed for the other low-DIN FFPE samples in order to get a library pool to be sequenced. A DNA library using 10 cycles pre-hyb PCR was also constructed for NA12892 control sample for detection of possible negative impact on the results.

**Fig. 4:**
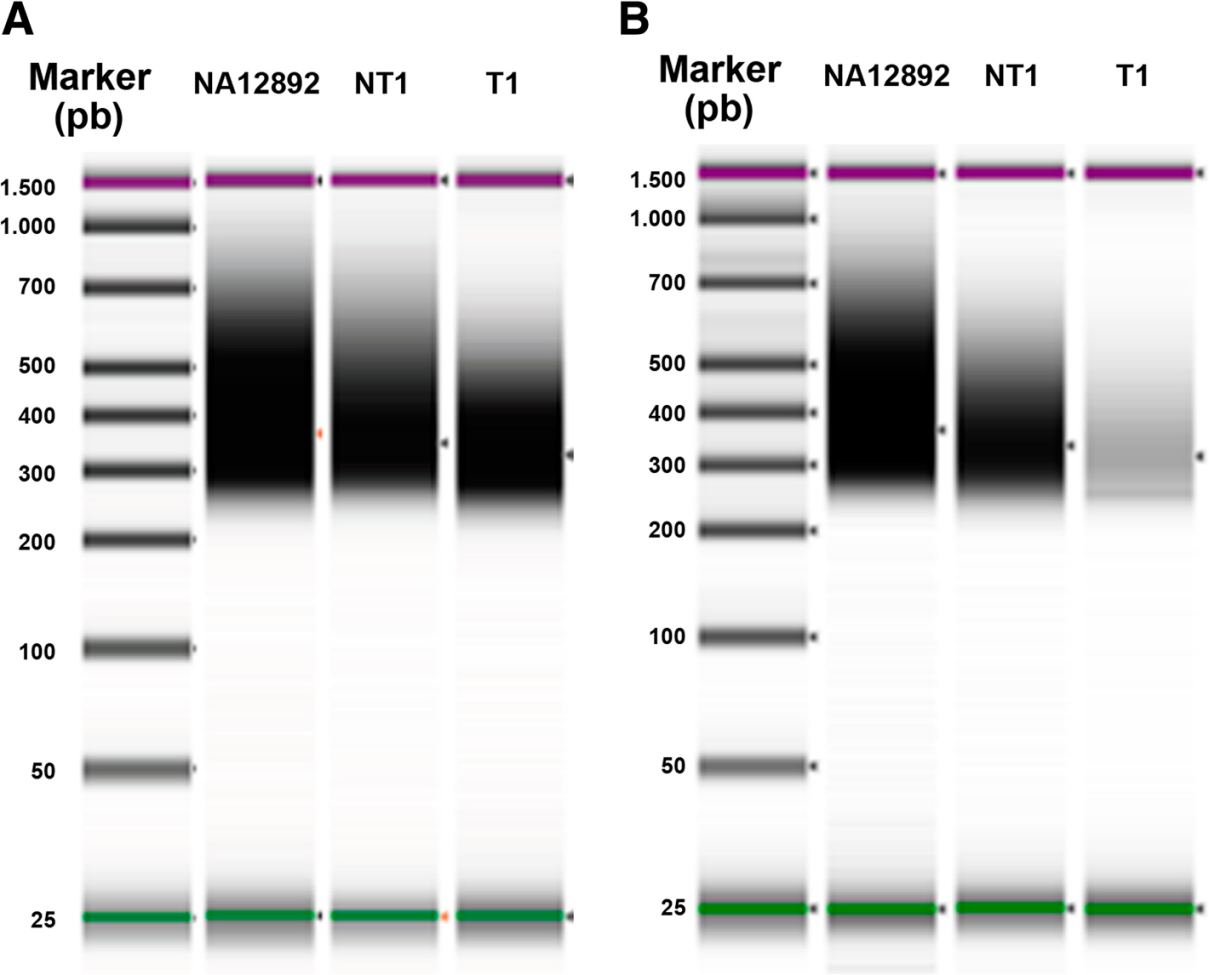
Gel image of NA12892, NT1 and T1 pre-hyb products with the Agilent 2200 Tape station and D1000 Screen Tape. Input DNA was 50 ng. **a** Using standard reagent volumes. **b** Using ½x standard reagent volumes

The results from the first sequencing run (see Table 1) confirmed that only a small quantity of final library was necessary to achieve satisfactory sequencing results. The behavior of high-quality DNA controls was similar to those reported in previous studies [19, 20]. We then tested the impact of using half of the originally recommended reagent volumes. We assumed that, in high-quality DNA samples, the impact should not be significant, however in low-quality DNA samples, keeping 50 ng of DNA input in half the volume could help increasing the variety of available fragments, due to increased DNA concentration. Figure 4 shows the pre-hyb fragment profiles for NA12892, NT1 and T1, using 1× or $$ \raisebox{1ex}{$1$}\!\left/ \!\raisebox{-1ex}{$2$}\right. $$x reagent volumes. As expected, impact on the concentration for high-quality DNA samples (NA12892 and NT1) was low, while the impact was remarkable for T1. We also tested the impact of using a quarter of the originally recommended reagent volumes. Results showed a drastic decrease of the concentration (up to more than 10 times), especially noticeable in HQ samples (see Additional file 1: Figure S1). In addition, we found that using a quarter of the volumes could risk the library preparation in different steps. Therefore, we kept using half of the originally recommended volumes.

**Table 1:** Results from the first sequencing run

Sample	DIN	#Pre-hyb Cycles	Hyb Input (ng)	#Sequences	Aligned	On target	No PCR Dup	Final	MedianDepth (per million reads)
NA12878	9.3	8	750	3,387,238	99.37%	75.07%	48.16%	48.14%	113.07
NA12891	9.1	8	750	3,188,127	99.48%	72.49%	49.56%	49.54%	118.25
NA12892	9.1	8	750	3,115,861	98.88%	73.80%	50.41%	50.40%	119.07
NT1	6.4	8	401	11,305,259	99.72%	71.49%	31.15%	31.13%	63.42
T1	2.7	10	362.5	2,866,206	99.37%	72.20%	35.17%	35.13%	66.29
T2	2.2	10	11,9	2,813,675	97.65%	81.07%	4.45%	4.40%	4.98
T3	3	10	87	2,730,937	99.39%	77.30%	13.86%	13.84%	25.63
T4	2	10	12	2,347,778	97.87%	71.73%	5.09%	5.05%	7.24
T5	3.6	10	32.4	3,077,225	99.35%	79.63%	8.45%	8.42%	13.65
NA12892	9.1	10	156	2,983,904	98.63%	77.31%	49.21%	49.19%	106.24

From left to right: sample name, DIN value for input DNA, number of cycles in the pre-hyb PCR, total amount of digested and adaptor-tagged library used in the hybridization step, number of sequences obtained, percentage of sequences aligned to the human reference genome, percentage of sequences on target, percentage of sequences after PCR duplicates removal, percentage of sequences after low-quality-mapped-reads removal, median of the depth in the target regions obtained per million reads (normalized for a more realistic comparison)

**Fig. 5:**
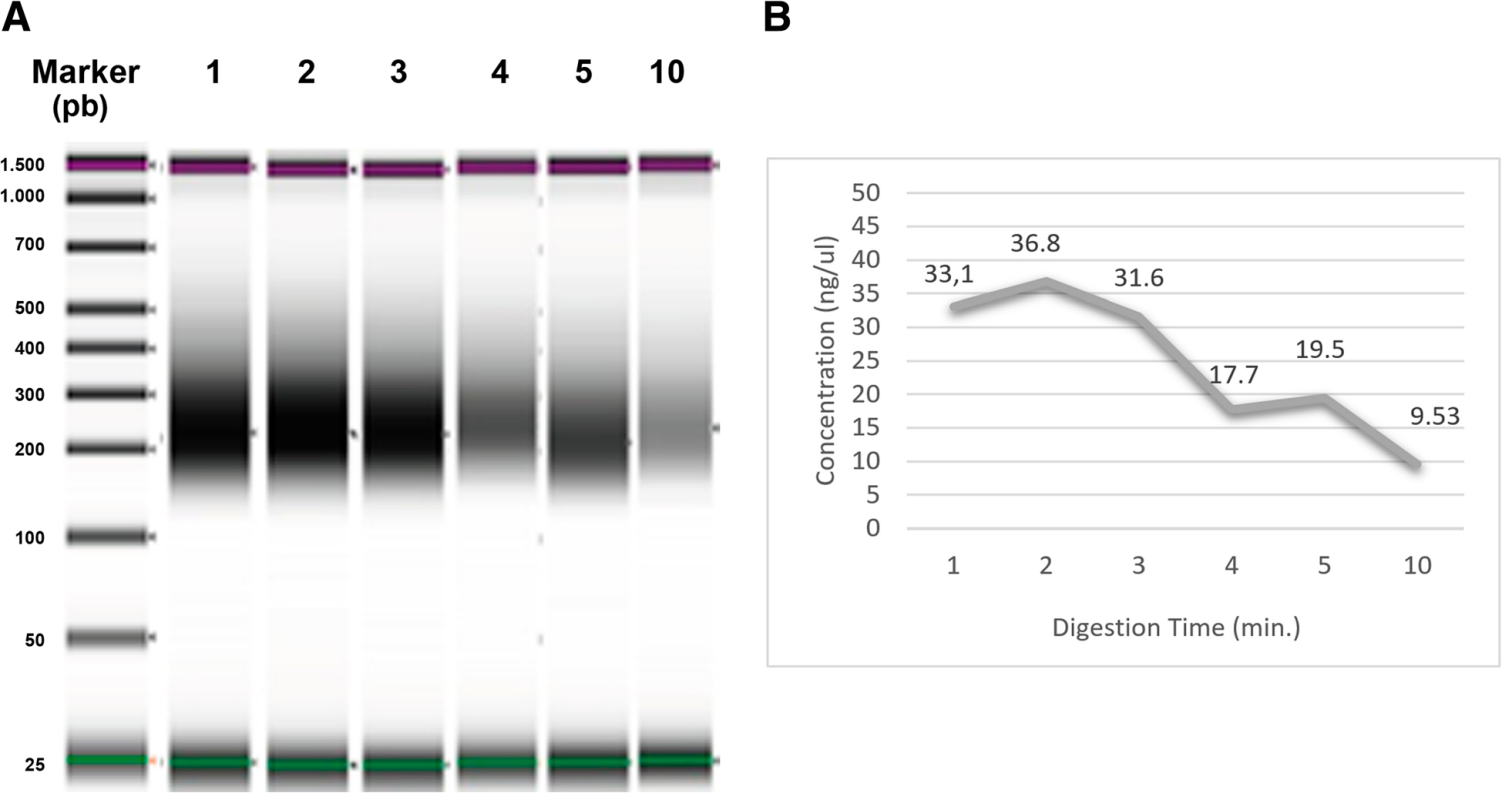
Pre-hyb library quantification using the Agilent 2200 Tape station and D1000 Screen Tape. **a** Gel image showing library products where digestion times were set up at 1, 2, 3, 4, 5 and the standard 10 min. **b** Graphical representation of pre-hyb library concentration vs. digestion times. **c** Gel image of different DNA input and digestion time tests. **b** DNA input and digestion time conditions and pre-hyb concentration

Next, we performed a series of tests focusing on the digestion time (since tranposases can work repeatedly, a shorter period of enzymatic activity could be beneficial for low-quality DNA-samples). Results (Fig. 5a and b) showed an increased in concentration of pre-hyb product. In parallel, we proceeded with those FFPE DNA cases which presented a good DIN (higher than 6). Increased digestion time was tested for different quantities of NT1 DNA, under the hypothesis that longer transposase activity may increase the final amount of digested fragments with desirable sizes. A final 150 ng of input DNA and a digestion time of 30 min gave optimal results (Fig. 5c and d) giving the highest pre-hyb concentration (521 nM). Nonetheless, when we applied these modifications to a sample with an intermediate DIN value of 4.6, pre-hyb concentration did not show any improvement compared to standard input (50 ng) and digestion time (1 min): 15.4 vs. 36.3 nM.

PCR duplicates have a very high impact in the sequencing results (see Table 1), thus we reduced the number of cycles in the post-hyb PCR to 10. In order to see how these modifications affect the library construction, we performed a second sequencing run (see Table 2). An increment in both the percentage of useful sequences and the median depth per million reads was noticeable for low-quality DNA samples. Finally, we included two other modifications: reduction of the amount of probes in order to reduce the capture of PCR-duplicate fragments in the hybridization step and also reduction of the number of cycles in the post-hyb PCR step to 8. A third set of sequencing data was obtained for which results are also shown in Table 2.

**Table 2:**
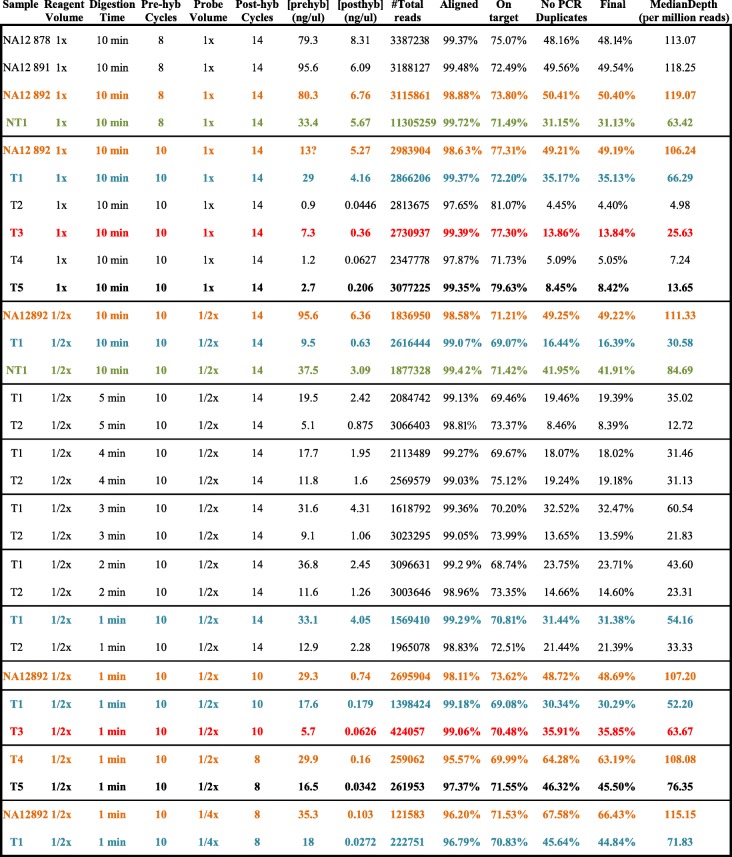
Global results from the 3 sequencing runs

From left to right: sample name, reagent volume (1× = standard, 1/2× = half standard), digestion time, number of cycles in pre-hyb PCR, volume of probe (1× = standard, 1/2× = half standard, 1/4× = quarter standard), number of cycles in post-hyb PCR, pre-hyb library concentration, post-hyb library concentration, number of sequences obtained, percentage of sequences aligned to human reference genome, percentage of sequences on target, percentage of sequences after PCR duplicates removal, percentage of sequences after low-quality-mapped-reads removal, median of the depth in target regions per million reads (normalized value for comparison). In orange, results for sample NA12892 highlighting that no negative impact was noticeable after modifications of the protocol in high-quality DNA samples. In green, improved results obtained for a high-quality FFPE sample (NT1) with some of the modification. In blue, results for sample T1 with notable improvement obtained after modifications. In red and bold, improved results for two very low-quality FFPE samples (T3 and T5) after modifications in the protocol

### DNA Quantification and Qualification

DNA samples extracted from FFPE tissues are known to present structural and molecular characteristics that can interfere with laboratory techniques. NGS techniques are especially sensitive to these characteristics. For this reason, we included a checkpoint for DNA quality as a previous step before the library construction. Although there may be some commercial QPCR alternatives, we consider this checkpoint both reliable and easy to perform. We used a pair of primers covering PTEN exon 1 region, previously validated [15], to quantitatively determine differences between DNA adequate for NGS and unusable FFPE DNA. Hence, we compared the amplification behaviour of two DNAs with decent DIN values (PNT1 = 6.4, PT1 = 4.6) and library preparation final concentration (PNT1 = 844 pM, PT1 = 299 pM). In order to to find out which condition highlighted the highest differences in quantification, we used decreasing numbers of amplification cycles (Additional file 2: Figure S2). According to our results, the highest [PTN1]/[PT1] value was obtained using 25 cycles (~ 6.5 times). Threfore, we chose 25 cycles to perform the PCR checkpoint.

**Fig. 6:**
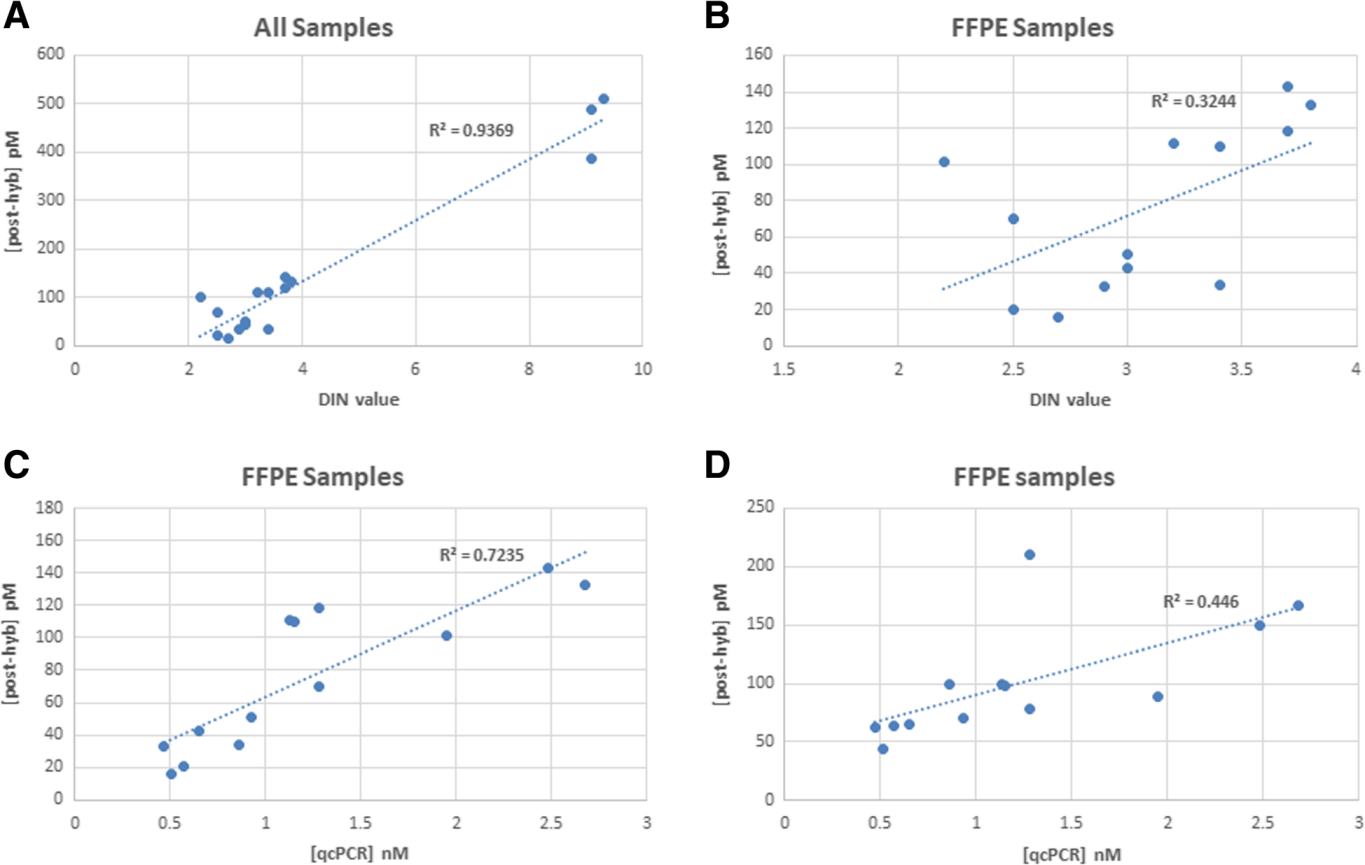
Correlations. **a** Correlation between post-hyb concentration measured by QPCR and DIN value for all samples. **b** Correlation between post-hyb concentration measured by QPCR and DIN value for FFPE samples. **c** Correlation between post-hyb concentration measured by QPCR and qcPCR concentration measured with Tape Station for FFPE samples. **d** Correlation between post-hyb concentration and qcPCR concentration measured, both with Tape Station, for FFPE samples

In order to estimate the correlation between PCR checkpoint values and final library concentration, libraries were constructed as indicated in the Modified SureSelect^QXT^ protocol for 16 different samples (see Additional file 3: Table S1). According to previous results during protocol modification, we considered acceptable a final library concentration > 50 pM obtained by QPCR. In addition, qcPCR checkpoint was also performed for all the 16 samples (qcPCR concentrations are also shown in Additional file 3: Table S1). We observed that the correlation between DIN value and final QPCR concentration was high (R^2^ = 0.94, Pearson *p*-value < 10^− 9^, see Fig. 6), probably due to differences between HQ and LQ samples. When HQ samples were removed, the correlation drastically decreased (R^2^ = 0.32, Pearson *p*-value = 0.042). Thus, DNA samples presenting a DIN value greater than two but lower than four constituted a heterogeneous group with an unclear behaviour during library preparation. On the other hand, correlation value between qcPCR concentration and final QPCR concentration was high (R^2^ = 0.72, Pearson *p*-value = 0.0002). Interestingly, correlation between qcPCR concentration and final library concentration measured with Tape Station was lower (R^2^ = 0.45, Pearson *p*-value = 0.01). We observed that Tape Station concentration measurement algorithm could be biased when high number of differences exists between sample concentrations (maximum and minimum concentrations were 738 and 43.8 pM respectively). We did not observe any correlation between age of the FFPE block and different library parameters. Finally, PCR checkpoint values ≤1 correlated with final QPCR concentrations under 50 pM, as can be observed in Table 3. Thus, stratification of samples with DIN values between two and four according to our qcPCR concentration predicted the behaviour of FFPE DNA samples during the SureSelect^QXT^ library preparation.

**Table 3:**
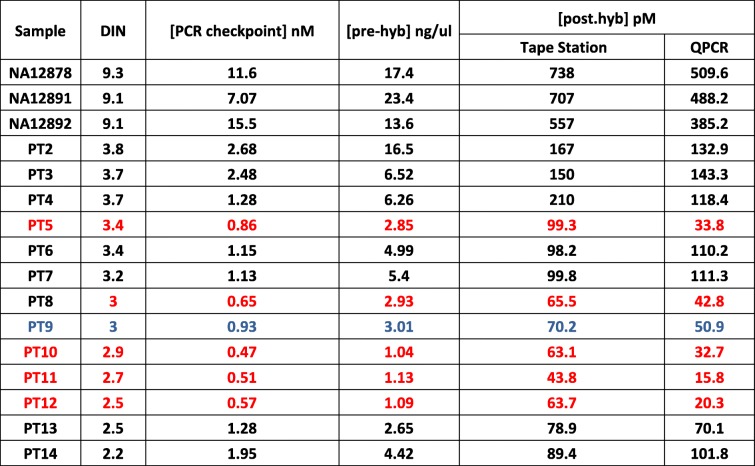
Descriptive of samples used for the validation of the qcPCR for FFPE DNA samples

From left to right: samples ID, DIN value, concentration of the PCR checkpoint, pre-hyb concentration, post-hyb concentration measured by Tape Station and QPCR. Highlighted in red those samples which would have been ruled out according to the checkpoint. Highlighted in blue a borderline sample

### Sample Pooling and Sequencing

The goal of sequencing tumour samples is to achieve enough read depth to reliably identify alterations that allow their molecular characterization. Consequently, library pool concentration is a crucial step for a successful sequencing process. It is recommended for Illumina Miseq platform to achieve 2 nM in order to avoid underclustering and hence poor results. However, achieving the concentration with libraries obtained using DNA extracted from FFPE samples and the SureSelectQ^XT^ protocol is often impossible. Therefore, we decided to perform a series of modifications which affect sample mixing and denaturing of sequencing pool prior to cluster generation.

Although equimolarity is desirable in all cases, our efforts were focused on prioritizing those library samples which showed lower concentrations, since the lack of read depth is a limiting factor. It is important to highlight that, on the other hand, an excess on read depth is not a problem. Thus, we performed two different sample mix strategies based on final library concentration: if the difference between the lowest concentrations and the average concentration was more than four times (Pool 1), we used all library preparation product for the lower concentrations and then we adjusted the rest of the samples to an equal concentration (Additional file 4: Table S3A). However, when concentrations were not so different (Pool 2), we mixed all the library preparation products from all the samples (Additional file 4: Table S3B). Since pool volumes were too large, a concentration step was performed, obtaining a final library concentration of 926.9 and 636.3 pM respectively for Pool 1 and Pool 2. According to manufacturer’s indications (see Methods section), final concentration for our library pools would have been 4.63 and 3.18 pM respectively. By reducing the HT1 Buffer volume to 600 μl, our theoretical final pool concentrations were 7.72 and 5.3 pM respectively for Pool 1 and Pool 2. Since minimum recommended pool concentration was 8 pM, we decided to sequence Pool 1. For Pool 2, we added another modification to the protocol: instead of adding 5 μl of 0.2 N NaOH solution to 5 μl of library pool, we added 1 μl of 1 N NaOH solution to 9 μl of library pool. Theoretical final library concentration for Pool 2 was 9.16 pM, which allowed us to complete the sequencing process. A summary of Pool 1 and Pool 2 features are shown in Additional file 4: Table S3. Differences observed between the percentages of reads obtained (column ratio average reads) and the percentage of reads expected (column ratio average conc.) might be due to a bias in the sequencing process based on different index efficiency or standard deviations of QPCR measures. Correlation between ratio average reads and ratio average concentration were 0.86 and 0.90 for Pool 1 and Pool 2 respectively (see Additional file 5: Figure S3). Observations from scatter plots highlight the efficiency of both sample mix strategies of sample stratification based on comparison to average final library concentration.

## Conclusions

The main conclusion from our study is that the behaviour of high quality DNA samples was completely different than the behaviour we observed for low quality FFPE DNA samples. This conclusion relays in different observations: the level of DNA degradation seemed to be critical, and for that reason, a higher amount of degraded DNA had a negative impact on the library concentration probably because too many small DNA fragments were available for the transposases. Another observation is that while using half volume of reagents in high-quality DNA samples seemed a straight-forward strategy, more modifications were needed in order to get satisfactory results for low-quality DNA samples. Moreover, we observed that larger amounts of high quality DNA got better results with increased digestion times, while, on the other hand, low quality DNA required shorter digestion times. Taking into account all the observations from our study, we have modified the SureSelect^QXT^ protocol to optimize results for our low-quality FFPE DNA samples.

## Methods

The aim of this study was the optimization of the SureSelect^QXT^ protocol (https://www.agilent.com/cs/library/usermanuals/public/G9681-90000.pdf) for the use of FFPE samples, resulting in a complete protocol modified for this purpose (Additional file 6: File S1). We have divided the modifications into three blocks:

### Modifications to Library Preparation Protocol

#### Samples

We selected 9 different DNA samples to make the validation tests: NA12878, NA12891 and NA12892 as high quality DNA extracted from cell lines and with known genotypes and karyotypes; NT1 as control of high quality DNA extracted from a normal fallopian tube FFPE sample; T1, T2, T3, T4 and T5 as regular tumour FFPE samples with known somatic variants and CNVs. DNA was extracted using the QIAamp® DNA FFPE Tissue Kit (Qiagen, Ref: 56404) and DIN value for each of the 9 genomic samples was measured using a Tape Station 2200 (Agilent, Ref: G2965AA) and the Genomic DNA kit (Refs: 5067–5365 and 5067–5366).

#### Experiments

- In order to know if we could achieve a greater number of fragments with a suitable size in the step of “Fragment and adaptor-tag the genomic DNA samples”, we tested different DNA inputs for sample NT1.
- To obtain sequenceable libraries for those samples with lower DIN, we increased up to 10 the cycles at the pre-hybridization (pre-hyb) PCR program keeping standard library preparation conditions (50 ng of input DNA).
- We tested the impact of using either half or a quarter of the originally recommended reagent volumes for library preparation under the assumption that in high-quality DNA samples, the impact should not be significant, however in low-quality DNA samples, keeping 50 ng of DNA input in lower volumes could help increasing the variety of available fragments, due to increased DNA concentration.
- For low-quality DNA-samples, decreased fragmentation times were tested based on the hypothesis that, since tranposases can work repeatedly during digestion time, a shorter period of enzymatic activity could be beneficial.
- For high-quality DNA-samples, increased fragmentation times were tested based on the hypothesis that longer transposase activity may increase the final amount of digested fragments with desirable sizes.
- To reduce the percentage of PCR duplicates obtained after library sequencing, decreased numbers of cycles were tested in the post-hybridization (post-hyb) PCR program. In addition, a lower volume of probes (0.25 μl/sample) was used during the hybridization step.

In all necessary cases, after the “Amplify the adaptor-tagged DNA library” step, we assessed quantity and quality of the libraries using the Agilent 2200 Tape Station and D1000 Screen Tape (Refs: 5067–5582 and 5067–5583).

### Modifications to DNA Quantification and Qualification

#### Samples

In order to check the correlation between PCR checkpoint values and final library concentration, we used different samples: NA12878, NA12891 and NA12892 as high quality DNA; PTN1 and PT1 as controls of high quality DNA extracted from FFPE samples; PT2, PT3, PT4, PT5, PT6, PT7, PT8, PT9, PT10, PT11, PT12, PT13 and PT14 as lab standard FFPE DNA samples. DIN values ranged from 2.2 to 3.8 (see Additional file 3: Table S1). Samples with DIN values < 2 were directly ruled out.

#### PTEN Exon 1 quality control PCR (qcPCR)

We designed a pair of primers covering PTEN exon 1 region (see Additional file 7: Table S2A), which was validated and used to confirm and check somatic mutations in a set of FFPE tumour samples [15], assuring the reliability of PCR reagents and program (Additional file 7: Table S2B and C). We used decreasing numbers of amplification cycles to find out which showed the highest differences in quantification, performed using the Agilent 2200 Tape Station and D1000 Screen Tape. Library preparation was performed as indicated in the Modified SureSelectQXT protocol, and final concentration was calculated through QPCR (as described in the Modified SureSelectQXT protocol) and also through the Agilent 2200 Tape Station and D1000 Screen Tape.

### Modifications to Sample Pooling and Sequencing

#### Samples and sample pooling

Library constructions were obtained as indicated in our modified protocol for 30 DNA samples extracted from FFPE tumour tissues that passed the qcPCR checkpoint. Final library concentrations were obtained by QPCR (see Additional file 4: Table S3). The 30 samples were divided into two different pool mixes according to following rules:

- If the difference between the lowest concentrations and the average concentration was more than 4 times, we used all library preparation product for the lower concentrations and then we adjusted the rest of the samples to an equal concentration.
- If the difference was smaller, we mixed all the library preparation products from all the samples.

Final pool volume from sample mixes was approximated 100 μl, with an expected concentration much below that required for sequencing, according to manufacturer’s indications. Hence, we used a Savant SpeedVac Concentrator (Thermo Scientific) to concentrate our library pools until a final desirable volume of 15–20 μl. Final concentration was obtained by using QPCR, according to manufacturer’s indications.

#### Modifications to pre-sequencing mix preparation

According to the manufacturer, standard procedure to handle library pool prior to sequencing is:

- Dilute library pool to 2 nM if necessary
- Add 5 μl of 2 nM library pool to 5 μl of 0.2 N NaOH
- Add 990 μl HT1 buffer to the 10 μl denature pool to reach a final concentration of 10 pM

We decided to modify the standard procedure taking into account the following limitations:

- Minimum volume of HT1 Buffer should be 600 μl.
- Maximum NaOH final concentration should be 0.0025 N.
- Minimum final pool concentration should be 8 pM

First, HT1 Buffer volume was reduced to 600 μl, calculating the theoretical library concentration using standard formula:

$$ Ci\times Vi= Cf\times V\mathrm{f}; $$

being Ci the initial pool concentration, Vi the initial volume, Cf the final theoretical concentration and Vf the final volume.

If the Cf obtained was higher or close to 8 pM, the pool was considered suitable for sequencing. However, if the Cf obtained was lower than 8 pM, pool mix was obtained as follows:

- Add 1 μl of 1 N NaOH solution to 9 μl of library pool.
- Add 600 μl of HT1 buffer

#### NOTE

Sequencing of all the library pools was performed in a Miseq platform (Illumina) with paired-end protocol and v2 chemistry (300 cycles).

## Additional Files

Additional file 1: **Figure S1.** Quantification and qualification of the impact that using different reagent volumes (1×, 1/2× and 1/4×) had in the library preparation. A) NA12892 pre-hyb Tape Station D1000 fragment spectrum. B) NT1 pre-hyb Tape Station D1000 fragment spectrum. C) T1 pre-hyb Tape Station D1000 fragment spectrum. D) Table with pre-hyb and post-hyb concentration estimations. (DOCX 1259 kb)

Additional file 2: **Figure S2.** Quantification and qualification of PTEN Exon 1 PCR fragments with the Agilent 2200 Tape station and D1000 Screen Tape. A) Gel image. B) Table with conditions and concentration estimations. (DOCX 57 kb)

Additional file 3: **Table S1.** Descriptive of samples used for the validation of the qcPCR for FFPE DNA samples. From left to right: samples ID, DIN value, concentration of the PCR checkpoint, pre-hyb concentration, post-hyb concentration measured by Tape Station and QPCR. Highlighted in red those samples which would have been ruled out according to the checkpoint. Highlighted in blue a borderline sample. (DOCX 14 kb)

Additional file 4: **Table S3.** Sample pooling in two different sequencing runs according to final library concentration measured by QPCR. From left to right: sample number, final library concentration, ratio average concentration/library concentration, volume used for the pool, final estimated concentration in the pool, ratio average sample concentration in the pool/sample concentration. A) Sequencing run in which concentration variability was high. B) Sequencing run in which concentration variability was medium-low. (DOCX 15 kb)

Additional file 5: **Figure S3.** Scatter plots of ratio average conc. vs. ratio average reads. A) Pool 1. B) Pool 2. (DOCX 28 kb)

Additional file 6: Modified SureSelect^QXT^ Target Enrichment Protocol for Illumina Multiplexed Sequencing of FFPE samples. (PDF 861 kb)

Additional file 7: **Table S2.** A) PTEN exon 1 primers for qcPCR. B) Preparation of quality control PCR Reaction mix. C) Thermal cycler program for Quality Control PCR. In blue the finally chosen number of cycles. (DOCX 18 kb)
