# Supplementary material for: Modified SureSelectQXT Target Enrichment Protocol for Illumina Multiplexed Sequencing of FFPE Samples
Source: Biol Proced Online. 2018 Oct 12;20:19. doi: 10.1186/s12575-018-0084-7 (PMC6182866; doi:10.1186/s12575-018-0084-7)
Supplement: Supplementary file 1 — Figure S1. Quantification and qualification of the impact that using different reagent volumes (1×, 1/2× and 1/4×) had in the library preparation. A) NA12892 pre-hyb Tape Station D1000 fragment spectrum. B) NT1 pre-hyb Tape Station D1000 fragment spectrum. C) T1 pre-hyb Tape Station D1000 fragment spectrum. D) Table with pre-hyb and post-hyb concentration estimations. (DOCX 1259 kb) [file 12575_2018_84_MOESM1_ESM.docx]

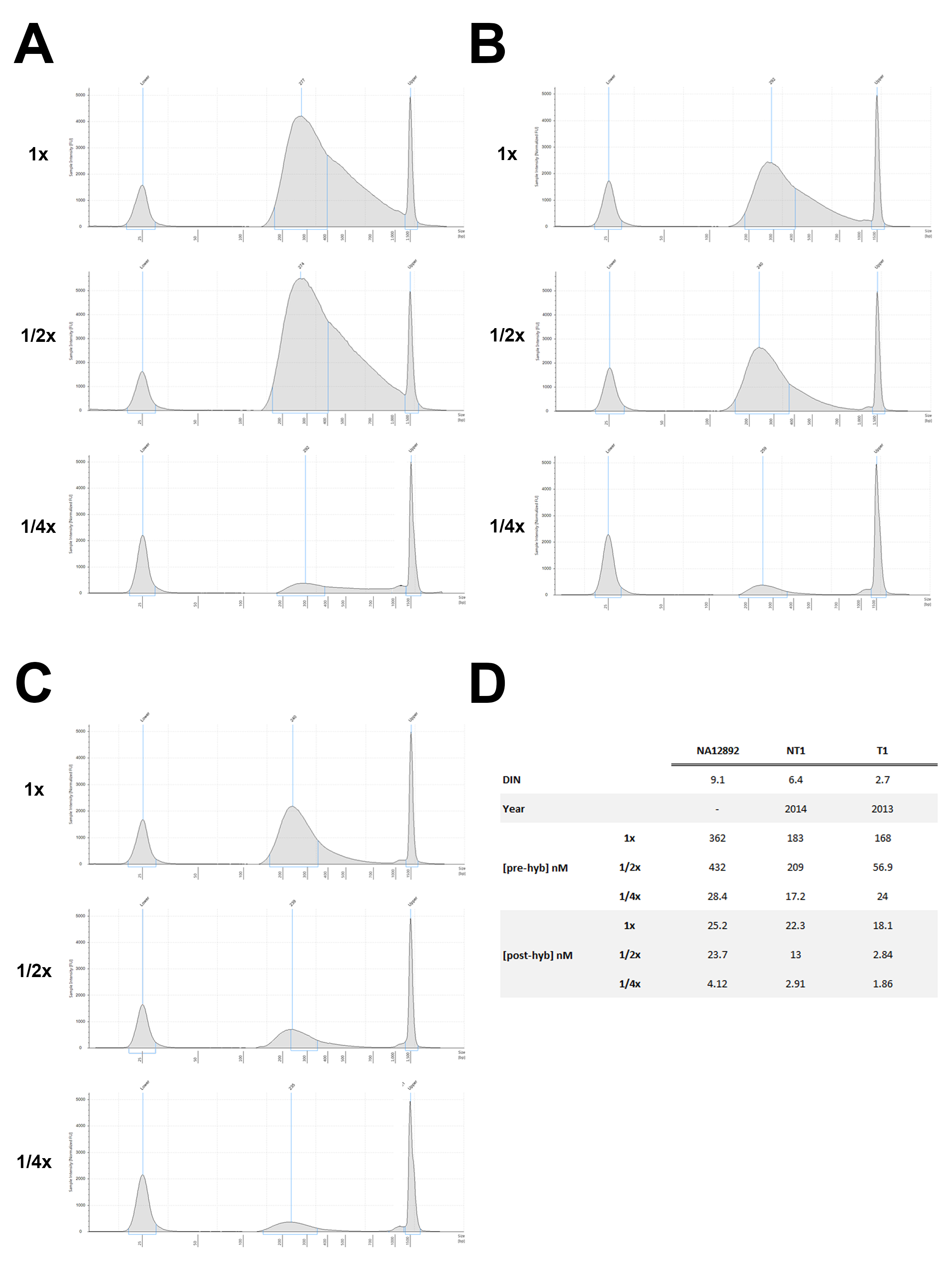


**Additional file 1: Figure S1:** Quantification and qualification of the impact that using different reagent volumes (1x, 1/2x and 1/4x) had in the library preparation. A) NA12892 pre-hyb Tape Station D1000 fragment spectrum. B) NT1 pre-hyb Tape Station D1000 fragment spectrum. C) T1 pre-hyb Tape Station D1000 fragment spectrum. D) Table with pre-hyb and post-hyb concentration estimations.
