# Supplementary material for: Modified SureSelectQXT Target Enrichment Protocol for Illumina Multiplexed Sequencing of FFPE Samples
Source: Biol Proced Online. 2018 Oct 12;20:19. doi: 10.1186/s12575-018-0084-7 (PMC6182866; doi:10.1186/s12575-018-0084-7)
Supplement: Supplementary file 2 — Figure S2. Quantification and qualification of PTEN Exon 1 PCR fragments with the Agilent 2200 Tape station and D1000 Screen Tape. A) Gel image. B) Table with conditions and concentration estimations. (DOCX 57 kb) [file 12575_2018_84_MOESM2_ESM.docx]

**A B**


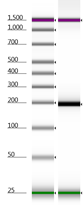

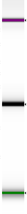

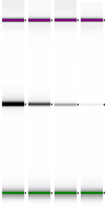


**1 2 3 4 5 6**

**Marker (pb)**

| **ID** | **Sample** | **#Cycles** | **Concentration (nM)** |
| --- | --- | --- | --- |
| 1 | PNT1 | 40 | 19.41 |
| 2 | PT1 |  | 17.83 |
| 3 | PNT1 | 30 | 15.90 |
| 4 | PT1 |  | 4.97 |
| 5 | PNT1 | 25 | 1.67 |
| 6 | PT1 |  | 0.26 |

**Additional file 2: Figure S2:** Quantification and qualification of PTEN Exon 1 PCR fragments with the Agilent 2200 Tape station and D1000 Screen Tape. A) Gel image. B) Table with conditions and concentration estimations.
