# Supplementary material for: Modified SureSelectQXT Target Enrichment Protocol for Illumina Multiplexed Sequencing of FFPE Samples
Source: Biol Proced Online. 2018 Oct 12;20:19. doi: 10.1186/s12575-018-0084-7 (PMC6182866; doi:10.1186/s12575-018-0084-7)
Supplement: Supplementary file 3 — Table S1. Descriptive of samples used for the validation of the qcPCR for FFPE DNA samples. From left to right: samples ID, DIN value, concentration of the PCR checkpoint, pre-hyb concentration, post-hyb concentration measured by Tape Station and QPCR. Highlighted in red those samples which would have been ruled out according to the checkpoint. Highlighted in blue a borderline sample. (DOCX 14 kb) [file 12575_2018_84_MOESM3_ESM.docx]

Additional file 3:Table S1: Descriptive of samples used for the validation of the qcPCR for FFPE DNA samples. From left to right: samples ID, DIN value, concentration of the PCR checkpoint, pre-hyb concentration, post-hyb concentration measured by Tape Station and QPCR. Highlighted in red those samples which would have been ruled out according to the checkpoint. Highlighted in blue a borderline sample.

| **Sample** | **Year** | **DIN** | **[PCR checkpoint] nM** | **[pre-hyb] ng/ul** | **[post.hyb] pM** | |
| --- | --- | --- | --- | --- | --- | --- |
|  |  |  |  |  | **Tape Station** | **QPCR** |
| **NA12878** | **-** | **9.3** | **11.6** | **17.4** | **738** | **509.6** |
| **NA12891** | **-** | **9.1** | **7.07** | **23.4** | **707** | **488.2** |
| **NA12892** | **-** | **9.1** | **15.5** | **13.6** | **557** | **385.2** |
| **PT2** | **2005** | **3.8** | **2.68** | **16.5** | **167** | **132.9** |
| **PT3** | **2007** | **3.7** | **2.48** | **6.52** | **150** | **143.3** |
| **PT4** | **2006** | **3.7** | **1.28** | **6.26** | **210** | **118.4** |
| **PT5** | **2010** | **3.4** | **0.86** | **2.85** | **99.3** | **33.8** |
| **PT6** | **2004** | **3.4** | **1.15** | **4.99** | **98.2** | **110.2** |
| **PT7** | **2007** | **3.2** | **1.13** | **5.4** | **99.8** | **111.3** |
| **PT8** | **1999** | **3** | **0.65** | **2.93** | **65.5** | **42.8** |
| **PT9** | **2010** | **3** | **0.93** | **3.01** | **70.2** | **50.9** |
| **PT10** | **2008** | **2.9** | **0.47** | **1.04** | **63.1** | **32.7** |
| **PT11** | **2006** | **2.7** | **0.51** | **1.13** | **43.8** | **15.8** |
| **PT12** | **2010** | **2.5** | **0.57** | **1.09** | **63.7** | **20.3** |
| **PT13** | **2005** | **2.5** | **1.28** | **2.65** | **78.9** | **70.1** |
| **PT14** | **2006** | **2.2** | **1.95** | **4.42** | **89.4** | **101.8** |
