# Supplementary material for: Modified SureSelectQXT Target Enrichment Protocol for Illumina Multiplexed Sequencing of FFPE Samples
Source: Biol Proced Online. 2018 Oct 12;20:19. doi: 10.1186/s12575-018-0084-7 (PMC6182866; doi:10.1186/s12575-018-0084-7)
Supplement: Supplementary file 4 — Table S3. Sample pooling in two different sequencing runs according to final library concentration measured by QPCR. From left to right: sample number, final library concentration, ratio average concentration/library concentration, volume used for the pool, final estimated concentration in the pool, ratio average sample concentration in the pool/sample concentration. A) Sequencing run in which concentration variability was high. B) Sequencing run in which concentration variability was medium-low. (DOCX 15 kb) [file 12575_2018_84_MOESM4_ESM.docx]

**Additional file 4: Table S3:** Sample pooling in two different sequencing runs according to final library concentration measured by QPCR. From left to right: sample number, final library concentration, ratio average concentration / library concentration, volume used for the pool, final estimated concentration in the pool, ratio average sample concentration in the pool / sample concentration. A) Sequencing run in which concentration variability was high. B) Sequencing run in which concentration variability was medium-low.

**A**

| **Sample** | **[library](pM)** | **ratio average** | **Vol. used (ul)** | **[Final](pM)** | **ratio average** |
| --- | --- | --- | --- | --- | --- |
| 1 | **67** | 3.76 | 8.5 | 5.7 | 2.00 |
| 2 | **124** | 2.03 | 8 | 10.0 | 1.15 |
| 3 | **148** | 1.70 | 8 | 11.9 | 0.96 |
| 4 | **458** | 0.55 | 3 | 13.8 | 0.83 |
| 5 | **237** | 1.06 | 5 | 11.9 | 0.96 |
| 6 | **162** | 1.55 | 8 | 13.0 | 0.88 |
| 7 | **143** | 1.76 | 8 | 11.5 | 0.99 |
| 8 | **824** | 0.31 | 2 | 16.6 | 0.69 |
| 9 | **118** | 2.13 | 8 | 9.5 | 1.20 |
| 10 | **442** | 0.57 | 3 | 13.3 | 0.86 |
| 11 | **265** | 0.95 | 5 | 13.3 | 0.86 |
| 12 | **150** | 1.68 | 8 | 12.1 | 0.95 |
| 13 | **487** | 0.52 | 3 | 14.7 | 0.78 |
| 14 | **260** | 0.97 | 5 | 13.1 | 0.87 |
| 15 | **53** | 4.75 | 8.5 | 4.5 | 2.52 |
| 16 | **92** | 2.74 | 8.5 | 7.9 | 1.45 |
|  |  |  |  |  |  |
| **Average** | 252 | 1.69 | 6.22 | 11.42 | 1.12 |

**B**

| **Sample** | **[library](pM)** | **ratio average** | **Vol. used (ul)** | **[Final](pM)** | **ratio average** |
| --- | --- | --- | --- | --- | --- |
| 1 | **69** | 1.27 | 8 | 4.9 | 1.27 |
| 2 | **136** | 0.64 | 8 | 9.7 | 0.64 |
| 3 | **80** | 1.10 | 8 | 5.7 | 1.10 |
| 4 | **74** | 1.19 | 8 | 5.3 | 1.19 |
| 5 | **39** | 2.25 | 8 | 2.8 | 2.25 |
| 6 | **41** | 2.14 | 8 | 2.9 | 2.14 |
| 7 | **72** | 1.22 | 8 | 5.1 | 1.22 |
| 8 | **148** | 0.59 | 8 | 10.6 | 0.59 |
| 9 | **105** | 0.84 | 8 | 7.5 | 0.84 |
| 10 | **50** | 1.75 | 8 | 3.6 | 1.75 |
| 11 | **125** | 0.70 | 8 | 8.9 | 0.70 |
| 12 | **84** | 1.04 | 8 | 6.0 | 1.04 |
| 13 | **85** | 1.03 | 8 | 6.1 | 1.03 |
| 14 | **120** | 0.73 | 8 | 8.6 | 0.73 |
|  |  |  |  |  |  |
| **Average** | 88 | 1 | 8 | 6 | 1 |
