# Supplementary material for: Modified SureSelectQXT Target Enrichment Protocol for Illumina Multiplexed Sequencing of FFPE Samples
Source: Biol Proced Online. 2018 Oct 12;20:19. doi: 10.1186/s12575-018-0084-7 (PMC6182866; doi:10.1186/s12575-018-0084-7)
Supplement: Supplementary file 5 — Figure S3. Scatter plots of ratio average conc. vs. ratio average reads. A) Pool 1. B) Pool 2. (DOCX 28 kb) [file 12575_2018_84_MOESM5_ESM.docx]

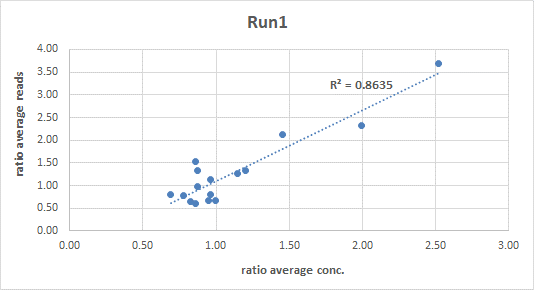

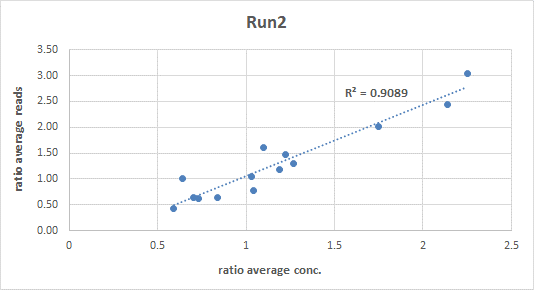
**A B**

**Additional file 5: Figure S3:** Scatter plots of ratio average conc. vs. ratio average reads. A) Pool 1. B) Pool 2.
