# Supplementary material for: Modified SureSelectQXT Target Enrichment Protocol for Illumina Multiplexed Sequencing of FFPE Samples
Source: Biol Proced Online. 2018 Oct 12;20:19. doi: 10.1186/s12575-018-0084-7 (PMC6182866; doi:10.1186/s12575-018-0084-7)
Supplement: Supplementary file 6 — Modified SureSelectQXT Target Enrichment Protocol for Illumina Multiplexed Sequencing of FFPE samples. (PDF 861 kb) [file 12575_2018_84_MOESM6_ESM.pdf]

Modified SureSelect^{QXT} Target Enrichment Protocol for Illumina Multiplexed Sequencing of FFPE samples

Featuring Transposase-Based Library Prep Technology

For Research Use Only. Not for use in diagnostic procedures.

This Protocol is based on the version D0, November 2015. Make sure you read and understand Agilent's protocol before using our modifications.

Contents

1. Overview of the Workflow 3

2. Sample Preparation 4

Step 1. Sample quality control checkpoint 5

Step 2. Fragment and adaptor-tag the genomic DNA samples 8

Step 3. Purify the adaptor-tagged library using AMPure XP beads 10

Step 4. Amplify the adaptor-tagged DNA library 11

Step 5. Purify the amplified library with AMPure XP beads 12

Step 6. Assess library DNA quantity and quality 13

3. Hybridization and Capture 14

Step 1. Hybridize DNA samples to the Capture Library 15

Step 2. Prepare streptavidin-coated magnetic beads for DNA hybrid capture 18

Step 3. Capture the hybridized DNA using streptavidin-coated beads 19

4. Indexing and Sample Processing for Multiplexed Sequencing 21

Step 1. Amplify the captured libraries to add index tags 22

Step 2. Purify the amplified captured libraries using AMPure XP beads 24

Step 3. Assess indexed library DNA quantity and quality 25

Step 4. Quantify each index-tagged library by QPCR 26

Step 5. Pool samples for multiplexed sequencing 27

Step 6. Prepare samples for sequencing 28

Nucleotide Sequences of SureSelect^{QXT} Dual Indexes 30

Guidelines for Multiplexing with Dual-Indexed Samples 32

1. Overview of the Workflow

The SureSelect^{QXT} target enrichment workflow is summarized in Figure 1.

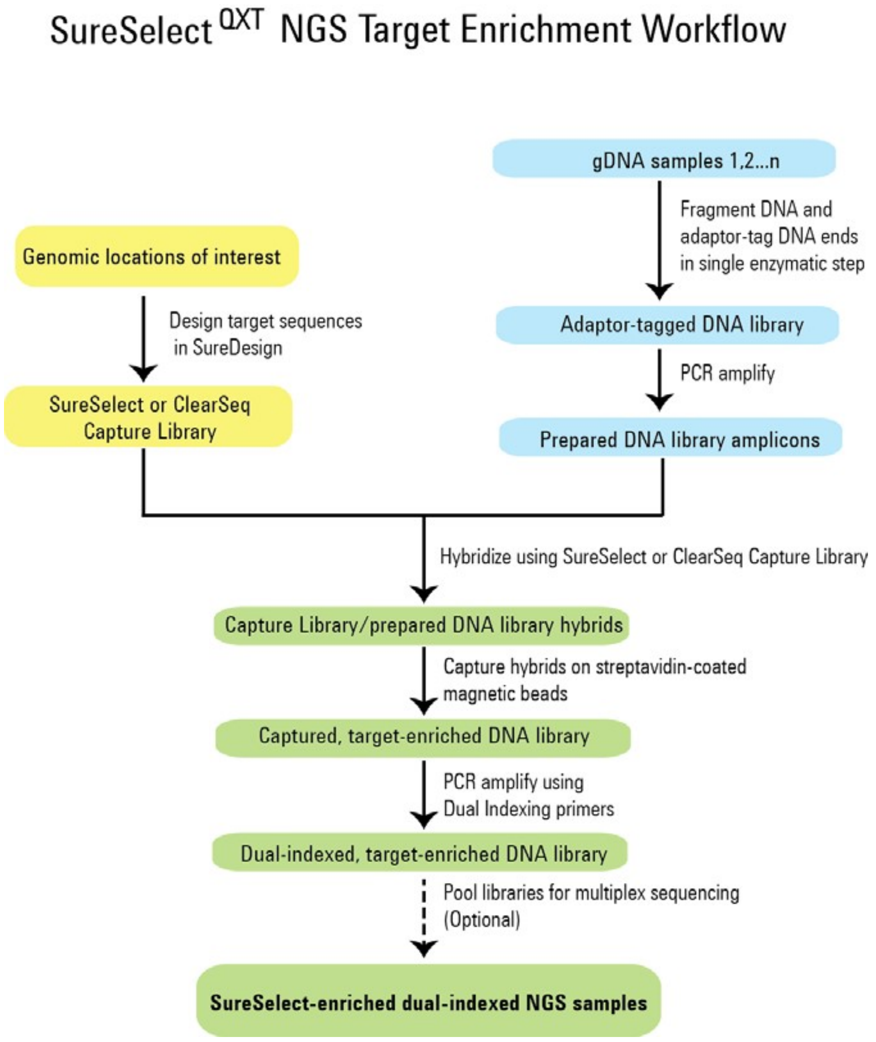

Figure 1: Overall target-enriched sequencing sample preparation workflow.

2. Sample Preparation

Step 1. Sample quality control checkpoint 5

Step 2. Fragment and adaptor-tag the genomic DNA samples 8

Step 3. Purify the adaptor-tagged library using AMPure XP beads 10

Step 4. Amplify the adaptor-tagged DNA library 11

Step 5. Purify the amplified library with AMPure XP beads 12

Step 6. Assess library DNA quantity and quality 13

This section contains instructions for preparation of genomic DNA sequencing libraries prior to target enrichment, for subsequent sequencing on Illumina platforms.

Step 1. Sample quality control checkpoint

DNA from FFPE samples are usually degraded and fragmented, thus quantification and qualification of samples are crucial for the success of the protocol. In this regard, a quality control process has been included previous to the library generation. Decision tree workflow is summarized in Figure 2.

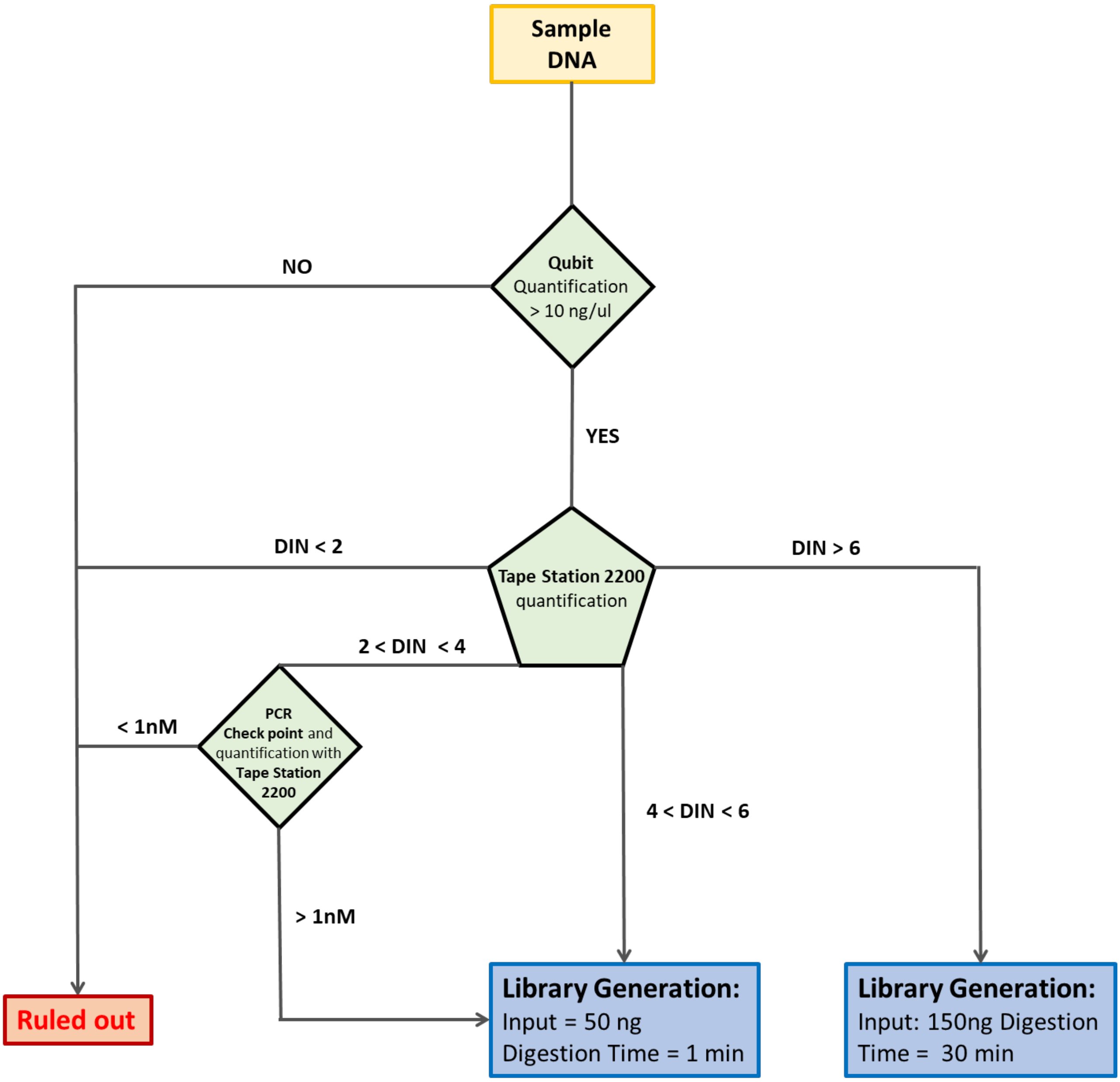

Figure 2: Decision tree for library preparation based on DNA quality.

a Use the Qubit dsDNA BR Assay or Qubit dsDNA HS Assay to determine the initial concentration of each gDNA sample. Follow the manufacturer's instructions for the specific assay kit and the Qubit instrument. This step is critical for successful preparation of input DNA.

b Use Tape Station 2200 using the Genomic DNA ScreenTape kit to determine DIN value of each gDNA sample, according to manufacturer's instructions.

c Dilute or concentrate the DNA samples to achieve 20 ng/ul. A minimum of 100 ng of DNA is required for the quality control PCR, although we strongly recommend an input of 200 ng of DNA.

d Using the primers described in Table 1, prepare the mix as presented in Table 2.

Table 1: PTEN exon 1 primers for quality control PCR

Name	Orientation	Sequence
PTEN_EX1_F	Forward	5´- GCAGCTTCTGCCATCTCTCT -3´
PTEN_EX1_R	Reverse	5´- CATCCGTCTACTCCCACGTT - 3´

e Set up the quality control reactions using a PCR plate or strip tube. Add 15 µl of the PCR mix previously prepared to each sample well.

Table 2: Preparation of quality control PCR Reaction mix

Reagent	Volume for 1 reaction	Volume for 16 reactions (includes excess)
Nuclease-free water	4.1 µl	69.7 µl
MgCl 25 mM*	1.5 µl	25.5 µl
10X PCR Buffer II*	2.5 µl	42.5 µl
dNTP mix 1,25mM**	4 µl	68 µl
10 µlM PTEN_EX1_F	1.25 µl	21.25 µl
10 µlM PTEN_EX1_R	1.25 µl	21.25 µl
AmpliTaq Gold DNA Polymerase (5 U/µL)*	0.4 µl	6.8 µl
**Total**	**15 µl**	**255 µl**

* From the kit AmpliTaq Gold^{TM} Polymerase with Buffer II and $MgCl_2$, Applied Biosystems^{TM}, ref. N8080247.
** Promega Corporation ref. U1240. To achieve required concentration, mix 12.5 µl from each of the 4 DNTPs at 100mM, then add 950 µl of nuclease free water.

f Add 10 µl of each DNA sample to its assigned sample well. While dispensing the DNA, be sure to place the pipette tip at the bottom of the well.

g Run the program (with the heated lid ON) in Table 3.

h For each sample, perform a quality control step in a Tape Station 2200 using the D1000 ScreenTape kit according to manufacturer's instructions.

Table 3: Thermal cycler program for Quality Control PCR

Segment Number	Number of Cycles	Temperature	Time
1	1	94°C	6 minutes
2	25	94°C	45 seconds
		54°C	45 seconds
		72°C	1 minute
3	1	72°C	7 minutes
4	1	4°C	Hold

Step 2. Fragment and adaptor-tag the genomic DNA samples

In this step, the gDNA is enzymatically fragmented and adaptors are added to ends of the fragments in a single reaction.

Before you begin, remove the SureSelect QXT Enzyme Mix ILM and the SureSelect QXT Buffer tubes from storage at –20°C and place on ice. Vortex each reagent vigorously to mix before use. Remove the AMPure XP beads from storage at 4°C and allow to warm up to room temperature.

NOTE While obtaining components for this step, also remove the DMSO vial from the SureSelect QXT Library Prep Kit Box 2 in –20°C storage. Leave the DMSO vial at room temperature in preparation for use on page 11.

For each DNA sample to be sequenced, prepare 1 library.

1 Verify that the SureSelect QXT Stop Solution contains 25% ethanol, by referring to the container label and the instructions below.
Before the first use of a fresh container, add 1.5 ml of ethanol to the provided bottle containing 4.5 ml of stop solution, for a final ethanol concentration of 25%. Seal the bottle then vortex well to mix. After adding the ethanol, be sure to mark the label for reference by later users.
Keep the prepared 1X SureSelect QXT Stop Solution at room temperature, tightly sealed, until it is used on page 9.

2 Prepare reagents for the purification protocols on page 10 and page 12.

a Transfer the AMPure XP beads to room temperature. The beads should be held at room temperature for at least 30 minutes before use. *Do not freeze the beads at any time*.
b Prepare 800 µl of fresh 70% ethanol per sample, plus excess, for use in the purification steps. The 70% ethanol may be used for multiple steps done on the same day, when stored in a sealed container.

CAUTION The duration and temperature of incubation for DNA fragmentation must be precisely controlled for optimal results. Make sure to preprogram the thermal cycler as directed in step 3 before setting up the fragmentation reactions. **Do not exceed the time at 45°C**, as indicated in Table 4.

Make sure you group the samples according to Digestion Time based on quality control results!

3 Preprogram a thermal cycler (with the heated lid ON) with the program in Table 4. Start the program, then immediately press the *Pause* button, allowing the heated lid to reach temperature while you set up the fragmentation reactions.

Table 4: Thermal cycler program for DNA fragmentation

Step	Temperature	Time
Step 1	45°C	1 or 30 minutes
Step 2	4°C	1 minute
Step 3	4°C	Hold

4 Before use, vortex the SureSelect QXT Buffer and SureSelect QXT Enzyme Mix ILM tubes vigorously at high speed. Note that the SSEL QXT Buffer is viscous and thorough and vigorous mixing is critical for optimal fragmentation.

These components are in liquid form when removed from –20°C storage and should be returned to –20°C storage promptly after use in step 5.

CAUTION Minor variations in volumes of the solutions combined in step 5 below may result in DNA fragment size variation.

The SureSelect QXT Buffer and Enzyme Mix solutions are highly viscous. Be sure to follow the dispensing and mixing instructions in the steps below. Thorough mixing of the reagents and reactions is critical for optimal performance.

---

5 Set up the fragmentation reactions on ice using a PCR plate or strip tube. Components must be added in the order listed below. Do not pre-mix the SureSelect QXT Buffer and Enzyme Mix.

- **a** To each sample well, add 8.5 µl of SureSelect QXT Buffer.
- **b** Add 1 µl of each DNA sample (50 or 150 ng) to its assigned sample well. While dispensing the DNA, be sure to place the pipette tip at the bottom of the well.
- **c** Add 1 µl of SureSelect QXT Enzyme Mix, ILM to each sample well. While dispensing the enzyme mixture, place the pipette tip at the bottom of the well. After dispensing of the 1 µl of enzyme mix, pipette up and down 8 to 10 times to ensure complete transfer of the viscous solution to the well.

6 Seal the wells, briefly spin, and then **mix thoroughly by vortexing the plate or strip tube at high speed for 20 seconds**.

7 Briefly spin the samples, then immediately place the plate or strip tube in the thermal cycler. Press the *Play* button to resume the thermal cycling program in Table 4.

8 During the incubation of samples in the thermal cycler, vigorously vortex the AMPure XP beads at high speed to ensure homogeneous distribution of beads throughout the solution so that the beads are ready for use on page 10.

9 When the thermal cycler has completed the 1-minute incubation at 4°C, immediately place the samples on ice and proceed to step 10.

10 Add 16 µl of 1X SureSelect QXT Stop Solution (containing 25% ethanol) to each fragmentation reaction. Seal the wells with fresh caps, then vortex at high speed for 5 seconds. Briefly spin the plate or strip tube to collect the liquid.

Incubate the samples at room temperature for 1 minute. Proceed directly to the purification protocol on page 10.

Step 3. Purify the adaptor-tagged library using AMPure XP beads

Before you begin, verify that the AMPure XP beads have been incubated at room temperature for at least 30 minutes and that fresh 70% ethanol has been prepared for use in step 6.

1 Verify that the AMPure XP bead suspension has been well mixed and appears homogeneous and consistent in colour.

2 Add 26 µl of the homogeneous bead suspension to each well containing the DNA samples. Seal the wells with fresh caps, then vortex for 5 seconds. Briefly spin the samples to collect the liquid, without pelleting the beads.

Check that the beads are in a homogeneous suspension in the sample wells. Each well should have a uniform colour with no layers of beads or clear liquid present.

3 Incubate samples for 5 minutes at room temperature.

4 Put the plate or strip tube on the magnetic stand at room temperature. Wait for the solution to clear (approximately 3 to 5 minutes).

5 While keeping the plate or tubes in the magnetic stand, carefully remove and discard the cleared solution from each well. Do not disturb the beads while removing the solution.

6 Continue to keep the plate or tubes in the magnetic stand while you dispense 100 µl of fresh 70% ethanol in each sample well.

7 Wait for 1 minute to allow any disturbed beads to settle, then remove the ethanol.

8 Repeat step 6 and step 7 once for a total of two washes. Make sure to remove all of the ethanol at each wash step.

9 Dry the samples on the thermal cycler (with lid open) at 37°C for 1 to 3 minutes. Do not overdry the samples.

10 Add 5.5 µl of nuclease-free water to each sample well.

11 Seal the sample wells with fresh caps, then mix well on a vortex mixer and briefly spin the plate or tubes to collect the liquid.

12 Incubate for 2 minutes at room temperature.

13 Put the plate or tubes in the magnetic stand and leave for 2 minutes or until the solution in each well is clear.

14 Remove each cleared supernatant (approximately 5 µl) to wells of a fresh plate or strip tube and keep on ice. You can discard the beads at this time.

Step 4. Amplify the adaptor-tagged DNA library

In this step, the adaptor-tagged gDNA library is repaired and PCR-amplified.

1 Thaw then vortex to mix the reagents listed in Table 5. Keep all reagents except DMSO on ice.

Table 5: Preparation of pre-capture PCR Reaction mix

Reagent	Volume for 1 reaction	Volume for 16 reactions (includes excess)
Nuclease-free water	12.5 µl	212.5 µl
Herculase II 5× Reaction Buffer	5 µl	85 µl
100 mM dNTP Mix (25 mM each dNTP)	0.25 µl	4.25 µl
DMSO	1.25 µl	21.25 µl
SureSelect QXT Primer Mix	0.5 µl	8.5 µl
Herculase II Fusion DNA Polymerase	0.5 µl	8.5 µl
**Total**	**20 µl**	**340 µl**

2 Prepare the appropriate volume of PCR reaction mix, as described in Table 5, on ice. Mix well on a vortex mixer.

3 Add 19.5 µl of the pre-capture PCR reaction mix prepared in step 2 to each 5 µl purified DNA library sample.

Seal the wells with fresh caps and mix by vortexing gently for 5 seconds. Spin samples briefly to collect the liquid.

4 Incubate the plate in the thermal cycler (with the heated lid ON) and run the program in Table 6.

Table 6: Thermal cycler program for pre-capture PCR

Segment Number	Number of Cycles	Temperature	Time
1	1	68°C	2 minutes
2	1	98°C	2 minutes
3	**10**	98°C	30 seconds
		57°C	30 seconds
		72°C	1 minute
4	1	72°C	5 minutes
5	1	4°C	Hold

Step 5. Purify the amplified library with AMPure XP beads

Before you begin, verify that the AMPure XP beads have been kept at room temperature for at least 30 minutes and that fresh 70% ethanol has been prepared for use in step 5.

1 Mix the AMPure XP bead suspension well so that the suspension appears homogeneous and consistent in colour.

2 Transfer the samples to room temperature, then add 25 µl of the homogeneous bead suspension to each sample well containing the 25 µl amplified DNA samples. Seal the wells with fresh caps, then vortex for 5 seconds. Briefly spin the samples to collect the liquid without pelleting the beads. Check that the beads are in a homogeneous suspension in the sample wells. Each well should have a uniform colour with no layers of beads or clear liquid present. **Incubate samples for 5 minutes at room temperature**.

3 Put the plate or strip tube on the magnetic stand at room temperature. Wait for the solution to clear (approximately 3 to 5 minutes).

4 While keeping the plate or tubes in the magnetic stand, carefully remove and discard the cleared solution from each well. Do not disturb the beads while removing the solution.

5 Continue to keep the plate or tubes in the magnetic stand while you dispense 100 µl of fresh 70% ethanol in each sample well.

6 Wait for 1 minute to allow any disturbed beads to settle, then remove the ethanol.

7 Repeat step 6 and step 7 once for a total of two washes. Make sure to remove all of the ethanol at each wash step.

8 Dry the samples on the thermal cycler (with lid open) at 37°C for 1 to 3 minutes. Do not **overdry** the samples.

9 Add 7.5 µl of nuclease-free water to each sample well.

10 Seal the sample wells with fresh caps, then mix well on a vortex mixer and briefly spin the plate or tubes to collect the liquid.

11 Incubate for 2 minutes at room temperature.

12 Put the plate or tubes in the magnetic stand and leave for 2 minutes or until the solution in each well is clear.

13 Remove each cleared supernatant (approximately 7 µl) to wells of a fresh plate or strip tube. You can discard the beads at this time.

Stopping Point: If you do not continue to the next step, seal the wells and store the samples at 4°C short term or at –20°C for long term storage.

Step 6. Assess library DNA quantity and quality

Use a D1000 ScreenTape (p/n 5067-5582) and associated reagent kit (p/n 5067-5583) to analyze the amplified libraries using the Agilent 4200 TapeStation or 2200 TapeStation. For more information to do this step, see the appropriate TapeStation user manual at www.genomics.agilent.com.

1 Prepare the TapeStation samples as instructed in the instrument user manual. Use 1 µl of each amplified library DNA sample diluted with 3 µl of D1000 sample buffer for the analysis.

CAUTION Make sure that you thoroughly mix the combined DNA and sample buffer on a vortex mixer for 5 seconds for accurate quantitation.

2 Load the sample plate or tube strips from step 1, the D1000

ScreenTape, and loading tips into the TapeStation as instructed in the instrument user manual. Start the run.

3 Verify that the electropherogram shows the peak of DNA fragment size positioned between 245 to 325 bp. Sample electropherograms are shown in Figure 3. Variability of fragmentation profiles may be observed.

NOTE A peak DNA fragment size significantly less than 245 bp may indicate too little gDNA in the fragmentation reaction and may be associated with increased duplicates in the sequencing data. In contrast, a peak DNA fragment size significantly greater than 325 bp may indicate too much gDNA in the fragmentation reaction and may be associated with decreased percent-on-target performance in sequencing results.

4 Measure the concentration of each library by integrating under the entire peak.

Stopping Point If you do not continue to the next step, seal the library DNA sample plate and store at 4°C overnight or at –20°C for prolonged storage.

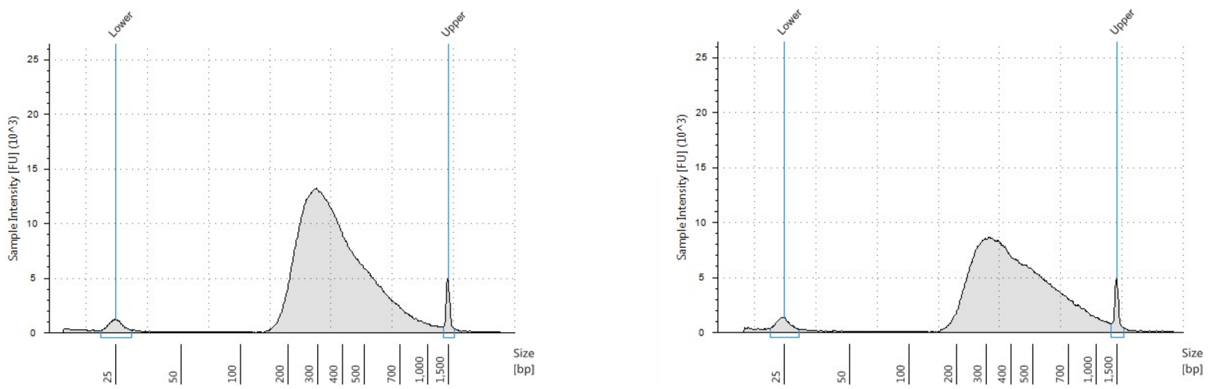

Figure 3: Representative sample electropherograms showing pre-capture analysis of amplified library DNA using the 2200 TapeStation with a D1000 ScreenTape.

3. Hybridization and Capture

Step 1. Hybridize DNA samples to the Capture Library 15

Step 2. Prepare streptavidin-coated magnetic beads for DNA hybrid capture 18

Step 3. Capture the hybridized DNA using streptavidin-coated beads 19

This chapter describes the steps to hybridize the prepared gDNA libraries with a target-specific Capture Library. After hybridization, the targeted molecules are captured on streptavidin beads.

CAUTION The ratio of Capture Library to gDNA library is critical for successful capture.

Step 1. Hybridize DNA samples to the Capture Library

This step uses the SureSelect^{QXT} Reagent Kit components listed in Table 7. Thaw each component under the conditions indicated in the table. Vortex each reagent to mix, then spin briefly to collect the liquid.

Table 7: Reagents for Hybridization and Capture

Kit Component	Storage Location	Thawing Conditions
SureSelect QXT Fast Hybridization Buffer	SureSelect QXT Hyb Module Box 2, –20°C	Warm to Room Temperature (RT), then keep at RT
SureSelect QXT Fast Blocker Mix	SureSelect QXT Hyb Module Box 2, –20°C	Thaw on ice
SureSelect RNase Block	SureSelect QXT Hyb Module Box 2, –20°C	Thaw on ice
Capture Library	–80°C	Thaw on ice

CAUTION For each protocol step that requires removal of tube cap strips, make sure to reseal the tubes with a fresh strip of domed caps. Reuse of strip caps can cause sample loss, sample contamination, or imprecision in sample temperatures during incubations.

1 To each adaptor-tagged DNA sample well, add 2.5 µl of SureSelect QXT Fast Blocker Mix. Pipette up and down 8 to 10 times to mix, then cap the wells. Vortex at high speed for 5 seconds, then spin the plate or strip tube briefly. Sample wells now contain 8.5 µl approx. of prepared DNA + Fast Blocker mixture.

2 Transfer the sealed prepared DNA + Blocker samples to the thermal cycler and start the following program shown in Table 8, using a heated lid.

Important: The thermal cycler must be paused during Segment 3 to allow additional reagents to be added to the Hybridization wells in step 5 on page 17.

During Segments 1 and 2 of the thermal cycling program, begin preparing the additional reagents as described in step 3 on page 16 and step 4 on page 16. If needed, you can finish these preparation steps after pausing the thermal cycler in Segment 3.

CAUTION The lid of the thermal cycler is hot and can cause burns. Use caution when working near the lid.

Table 8: Thermal cycler program for Hybridization*

Segment Number	Number of Cycles	Temperature	Time
1	1	95°C	5 minutes
2	1	65°C	10 minutes
3	1	65°C	1 minute **(PAUSE cycler here)**
4	60	65°C	1 minute
		37°C	3 seconds
5	1	65°C	Hold†

* When setting up the thermal cycling program, use a reaction volume setting of 30 uL (final volume of hybridization reactions during cycling in Segment 4).

3 Prepare a 25% solution of SureSelect RNase Block (containing 1 part RNase Block:3 parts water), according to Table 9. Prepare the amount required for the number of hybridization reactions in the run, plus excess. Mix well. Keep the stock vial and diluted RNase Block on ice.

Table 9: Preparation of RNase Block solution

Reagent	Volume for 1 reaction	Volume for 16 reactions (includes excess)	Volume for 96 reactions (includes excess)
SureSelect RNase Block	0.25 µl	5 µl	25 µl
Nuclease-free water	0.75 µl	15 µl	75 µl
**Total**	**1 µl**	**20 µl**	**100 µl**

NOTE Prepare the mixture described in step 4, below, just before pausing the thermal cycler in Segment 3 as described on above. It is important to prepare and keep the mixture at room temperature prior to addition to the DNA samples in step 5 on page 17. Solutions containing the Capture Library should not, however, be held at room temperature for extended periods.

4 Prepare the Capture Library Hybridization Mix appropriate for a capture library size < 3 Mb, as shown in Table 10.

Combine the listed reagents at room temperature. Mix well by vortexing at high speed for 5 seconds then spin down briefly. Proceed immediately to step 5.

Table 10: Preparation of Capture Library Hybridization Mix for **Capture Libraries < 3 Mb**

Reagent	Volume for 1 reaction	Volume for 16 reactions (includes excess)
25% RNase Block solution (from step 3)	1 µl	17 µl
Capture Library <3 Mb	0.5 µl	8.5 µl
SureSelect QXT Fast Hybridization Buffer	3 µl	51 µl
Nuclease-free water	2 µl	34 µl
**Total**	**6.5 µl**	**110.5 µl**

5 Once the thermal cycler starts Segment 3 of the program in Table 8 (1 minute at 65°C), press the *Pause* button. With the cycler paused, and while keeping the DNA + Blocker samples in the cycler, transfer 6 µl of the room-temperature Capture Library Hybridization Mix from step 4 to each sample well.

6 Mix well by pipetting up and down 8 to 10 times.

7 Seal the wells with fresh domed strip caps. Make sure that all wells are completely sealed. Vortex at high speed for 5 seconds, and then spin the tubes or plate briefly and return the samples to the thermal cycler. The hybridization reaction wells now contain approximately 14 µl.

8 Press the *Play* button to resume the thermal cycling program to allow hybridization of the prepared DNA samples to the Capture Library.

CAUTION

Wells must be adequately sealed to minimize evaporation, or your results can be negatively impacted.

Before you do the first experiment, make sure the plasticware and capping method are appropriate for the thermal cycler. Check that no more than 4 µl is lost to evaporation under the conditions used for hybridization.

Step 2. Prepare streptavidin-coated magnetic beads for DNA hybrid capture

1 Vigorously resuspend the Dynabeads MyOne Streptavidin T1 magnetic beads on a vortex mixer. The magnetic beads settle during storage.

2 For each hybridization sample, add 25 µl of the resuspended beads to wells of a fresh PCR plate or a strip tube.

3 Wash the beads:

- **a** Add 100 µl of SureSelect Binding Buffer.
- **b** Mix by pipetting up and down 10 times.
- **c** Put the plate or strip tube into a magnetic separator device.
- **d** Wait at least 5 minutes or until the solution is clear, then remove and discard the supernatant.
- **e** Repeat step a through step d two more times for a total of 3 washes.

4 Resuspend the beads in 100 µl of SureSelect Binding Buffer.

Step 3. Capture the hybridized DNA using streptavidin-coated beads

1 After the hybridization step is complete and the thermal cycler reaches the 65°C hold step (see Table 8 on page 16), transfer the samples to room temperature.

2 Maintain the hybridization samples at room temperature while you use a multichannel pipette to transfer the entire volume (approximately 14 µl) of each hybridization mixture to wells containing 100 µl of washed streptavidin beads. Seal the wells with fresh caps.

3 Incubate the capture plate or strip tube on a 96-well plate mixer, mixing vigorously (at 1800 rpm), for 30 minutes at room temperature.

Make sure the samples are properly mixing in the wells.

4 During the 30-minute incubation for capture, prewarm Wash Buffer 2 at 65°C as described below.

a Place 100 µl aliquots of Wash Buffer 2 in wells of a fresh 96-well plate or strip tubes. Aliquot 3 wells of buffer for each DNA sample in the run.

b Cap the wells with fresh domed caps and then incubate in the thermal cycler, with heated lid ON, held at 65°C until used in step 10.

5 When the 30-minute incubation period initiated in step 3 is complete, collect the liquid at the bottom of wells using the method appropriate for your labware:

- For samples in strip tubes, spin the samples briefly.
- For samples in 96-well plates, collect the liquid in the wells manually, using a swift, sharp flicking motion.

CAUTION Do not spin the streptavidin bead-bound DNA samples held in 96-well plates using a plate spinner or centrifuge during the wash steps. Spinning the 96-well plates can over-pack the streptavidin beads, preventing complete resuspension during the washes and can adversely impact sample recovery.

7 Put the plate or strip tube in a magnetic separator to collect the beads from the suspension. Wait 1 minute for the solution to clear, then remove and discard the supernatant.

8 Resuspend the beads in 100 µl of SureSelect Wash Buffer 1 (held at room temperature) by pipetting up and down 8 to 10 times. **Make sure the beads are in suspension before proceeding.**

9 Seal the wells with fresh caps, then mix by vortexing at high speed for 8 seconds. Collect the liquid at the bottom of wells using the method appropriate for your labware (spinning for strip tubes or manual collection for 96-well plates).

10 Put the plate or strip tube in a magnetic separator. Wait 1 minute for the solution to clear, then remove and discard the supernatant.

CAUTION Make sure that the Wash Buffer 2 is pre-warmed to 65°C before use in step 10 below.

11 **Remove the plate or strip tubes from the magnetic separator and transfer to a rack at room temperature.** Wash the beads with Wash Buffer 2, using the protocol steps below.

- **a** Resuspend the beads in 100 µl of 65°C prewarmed Wash Buffer 2. Pipette up and down at least 10 times to resuspend the beads.

  Make sure the beads are in suspension before proceeding.

- **b** Seal the wells with fresh caps and then vortex at high speed for 5 seconds. Collect the liquid at the bottom of wells using the method appropriate for your labware (spinning for strip tubes or manual collection for 96-well plates).

- **c** Incubate the samples for 10 minutes at 65°C on the thermal cycler with the heated lid on.

- **d** Put the plate or strip tube in the magnetic separator at room temperature.

- **e** Wait 1 minute for the solution to clear, then remove and discard the supernatant.

- **f** Repeat step a through step e two more times for a total of 3 washes.

12 After removing the supernatant from the final wash, spin the samples briefly, return the plate or tubes to the magnetic stand, and then remove any remaining wash buffer droplets.

13 Add 12 µl of nuclease-free water to each sample well. Place the capture plate or strip tube on ice until PCR reactions are set up on page x.

NOTE Captured DNA is retained on the streptavidin beads during the post-capture amplification step. Do not separate the supernatant from the beads at this step.

4. Indexing and Sample Processing for Multiplexed Sequencing

Step 1. Amplify the captured libraries to add index tags 22

Step 2. Purify the amplified captured libraries using AMPure XP beads 24

Step 3. Assess indexed library DNA quantity and quality 25

Step 4. Quantify each index-tagged library by QPCR (optional) 26

Step 5. Pool samples for multiplexed sequencing 27

Step 6. Prepare sequencing samples 28

This chapter describes the steps to add index tags by amplification, and to purify and assess quality and quantity of the indexed libraries. Sample pooling instructions are provided to prepare the indexed samples for multiplexed.

Step 1. Amplify the captured libraries to add index tags

In this step, the SureSelect-enriched DNA libraries are PCR amplified using the appropriate pair of dual indexing primers.

CAUTION To avoid cross-contaminating libraries, set up PCR mixes in a dedicated clean area or PCR hood with UV sterilization and positive air flow.

This step uses the components listed in Table 11. Thaw then vortex to mix the reagents listed below and keep on ice.

Table 11: Reagents for post-capture indexing by PCR amplification

Kit Component	Storage Location
Herculase II Fusion DNA Polymerase	SureSelect QXT Library Prep Kit Box 2, –20°C
Herculase II 5× Reaction Buffer	SureSelect QXT Library Prep Kit Box 2, –20°C
100 mM dNTP Mix (25 mM each dNTP)	SureSelect QXT Library Prep Kit Box 2, –20°C
SureSelect QXT P7 and P5 dual indexing primers	SureSelect QXT Library Prep Kit Box 2, –20°C

Prepare one indexing amplification reaction for each DNA library.

1 Determine the appropriate index assignments for each sample. See the Step 6. Prepare samples for sequencing section for sequences of the index portion of the P7 and P5 indexing primers used to amplify the DNA libraries in this step.

Use a different indexing primer combination for each sample to be sequenced in the same lane.

NOTE For sample multiplexing, Agilent recommends maximizing index diversity on both P7 and P5 primers as required for colour balance. For example, when 8-plexing, use eight different P7 index primers with two P5 index primers (see for Table 15 sequencing on MiSeq platform). See Table 16 and Table 17 on page 31 for additional details.

2 Prepare the appropriate volume of PCR reaction mix, as described in Table 12, on ice. Mix well on a vortex mixer.

Table 12: Preparation of post-capture PCR Reaction mix

Reagent	Volume for 1 reaction	Volume for 16 reactions (includes excess)
Nuclease-free water	6.75 µl	114.75 µl
Herculase II 5× Reaction Buffer	5 µl	85 µl
100 mM dNTP Mix (25 mM each dNTP)	0.25 µl	4.25 µl
Herculase II Fusion DNA Polymerase	0.5 µl	8.5 µl
**Total**	**12.5 µl**	**212.5 µl**

3 Obtain the plate or strip tube containing the bead-bound target-enriched DNA samples from ice. Add 12.5 µl of the PCR reaction mix prepared in step 2 to the 12 µl of bead suspension in each sample well.

4 Add 0.5 µl of the appropriate P7 dual indexing primer (P7 i1 to P7 i12) to each PCR reaction mixture well. Add only one of the twelve possible P7 primers to each reaction well.

5 Add 0.5 µl of the appropriate P5 dual indexing primer (P5 i13 to P5 i20) to each PCR reaction mixture well. Add only one of the eight possible P5 primers to each reaction well.

6 Mix well by pipetting to ensure the beads are fully resuspended, then transfer the PCR plate or strip tube to a thermal cycler and run the PCR amplification program shown in Table 13.

7 When the PCR amplification program is complete, spin the plate or strip tube briefly. Remove the streptavidin-coated beads by placing the plate or strip tube on the magnetic stand at room temperature. Wait 2 minutes for the solution to clear, then remove each supernatant (approximately 25 µl) to wells of a fresh plate or strip tube.

The beads can be discarded at this time.

Table 13: Post-Capture PCR cycling program

Segment	Number of Cycles	Temperature	Time
1	1	98°C	2 minutes
2	8	98°C	30 seconds
		58°C	30 seconds
		72°C	1 minute
3	1	72°C	5 minutes
4	1	4°C	Hold

Step 2. Purify the amplified captured libraries using AMPure XP beads

1 Let the AMPure XP beads come to room temperature for at least 0 minutes. *Do not freeze the beads at any time*.

2 Prepare 200 µl of fresh 70% ethanol per sample, plus excess, for use in step 8.

3 Mix the AMPure XP bead suspension well so that the suspension appears homogeneous and consistent in colour.

4 Add 30 µl of the homogeneous AMPure XP bead suspension to each 25 µl amplified DNA sample in the PCR plate or strip tube. Seal the wells with fresh caps, then vortex for 5 seconds. Briefly spin the samples to collect the liquid, without pelleting the beads.

Check that the beads are in a homogeneous suspension in the sample wells. Each well should have a uniform colour with no layers of beads or clear liquid present.

5 Incubate samples for 5 minutes at room temperature.

6 Put the plate or strip tube on the magnetic stand at room temperature. Wait for the solution to clear (approximately 3 to 5 minutes).

7 While keeping the plate or tubes in the magnetic stand, carefully remove and discard the cleared solution from each well. Do not disturb the beads while removing the solution.

8 Continue to keep the plate or tubes in the magnetic stand while you dispense 100 µl of fresh 70% ethanol in each sample well.

9 Wait for 1 minute to allow any disturbed beads to settle, then remove the ethanol.

10 Repeat step 8 and step 9 once for a total of two washes. Make sure to remove all of the ethanol at each wash step.

11 Dry the samples on the thermal cycler (with lid open) at 37°C for 1 to 3 minutes. Do not overdry the samples.

12 Add 14 µl of nuclease-free water to each sample well.

13 Seal the sample wells, then mix well on a vortex mixer and briefly spin the plate to collect the liquid.

14 Incubate for 2 minutes at room temperature.

15 Put the plate in the magnetic stand and leave for 2 minutes or until the solution is clear.

16 Remove the cleared supernatant (approximately 14 µl) to a fresh LoBind tube. You can discard the beads at this time.

Stopping Point: If you do not continue to the next step, store the libraries at –20°C.

Step 3. Assess indexed library DNA quantity and quality

Use a High Sensitivity D1000 ScreenTape (p/n 5067-5584) and reagent kit (p/n 5067-5585) to analyze the amplified indexed DNA using the Agilent 4200 TapeStation or 2200 TapeStation. For more information to do this step, see the appropriate TapeStation user manual at www.genomics.agilent.com.

1 Prepare the TapeStation samples as instructed in the instrument user manual. Use 2 µl of each indexed DNA sample diluted with 2 µl of High Sensitivity D1000 sample buffer for the analysis.

CAUTION Make sure that you thoroughly mix the combined DNA and sample buffer on a vortex mixer for 5 seconds for accurate quantitation.

2 Load the sample plate or tube strips from step 1, the High Sensitivity D1000 ScreenTape, and loading tips into the TapeStation as instructed in the instrument user manual. Start the run.

3 Verify that the electropherogram shows the peak of DNA fragment size positioned between 325 and 450 bp. A sample electropherogram is shown in Figure 4.

4 Measure the concentration of each library by integrating under the entire peak.

We strongly recommend to quantify the target enriched samples prior to pooling, proceed to “Step 4. Quantify each index-tagged library by QPCR (optional)” on page 26.

Stopping Point: If you do not continue to the next step, store the libraries at 4°C overnight or at –20°C for up to one month.

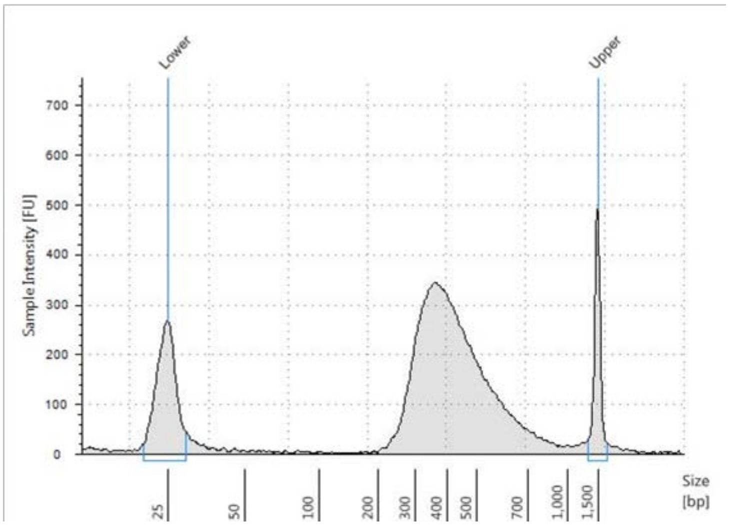

Figure 4: Post-capture analysis of amplified indexed library DNA using the 2200 TapeStation with a High Sensitivity D1000 ScreenTape.

Step 4. Quantify each index-tagged library by QPCR

Use the Agilent QPCR NGS Library Quantification Kit (for Illumina) to accurately determine the concentration of each index-tagged captured library. Refer to the protocol that is included with the Agilent QPCR NGS Library Quantification Kit (p/n G4880A) for more details to do this step.

1 Prepare a standard curve using the quantification standard included in the kit, according to the instructions provided in the user guide.

2 Dilute each index-tagged captured library such that it falls within the range of the standard curve. **For each sample, we recommend to adjust two dilutions between 10 and 1 pM based on the concentration obtained in the previous** "Step 3. Assess indexed library DNA quantity and quality."

3 Prepare the QPCR master mix with Illumina adaptor-specific PCR primers according to instructions provided in the kit.

4 Add an aliquot of the master mix to PCR tubes and add template.

5 On a QPCR system, **in our case LightCycler 480 II (Roche)**, run the thermal profile outlined in the QPCR NGS Library Quantification kit user guide. Use the SYBR Green instrument setting.

6 Use the standard curve to determine the concentration of each unknown index-tagged library, in nM.

Step 5. Pool samples for multiplexed sequencing

The number of indexed libraries that may be multiplexed in a single sequencing lane is determined by the output specifications of the platform used, together with the amount of sequencing data required for your research design. Calculate the number of indexes that can be combined per lane, according to the capacity of your platform and the amount of sequencing data required per sample.

Guidelines for optimal low-level multiplexing of samples indexed using the SureSelect^{QXT} dual indexes are provided on page 32.

Combine the libraries such that each index-tagged sample is present in equimolar amounts in the pool.

Step 6. Prepare samples for sequencing

We recommend a seeding concentration for SureSelect^{QXT} target-enriched libraries of **12** to **14 pM** on MiSeq instruments. Seeding concentration and cluster density may also need to be optimized based on the DNA fragment size range for the library and on the desired output and data quality. We recommend to follow the decision tree in Figure 5 in order to obtain successful results with FFPE samples.

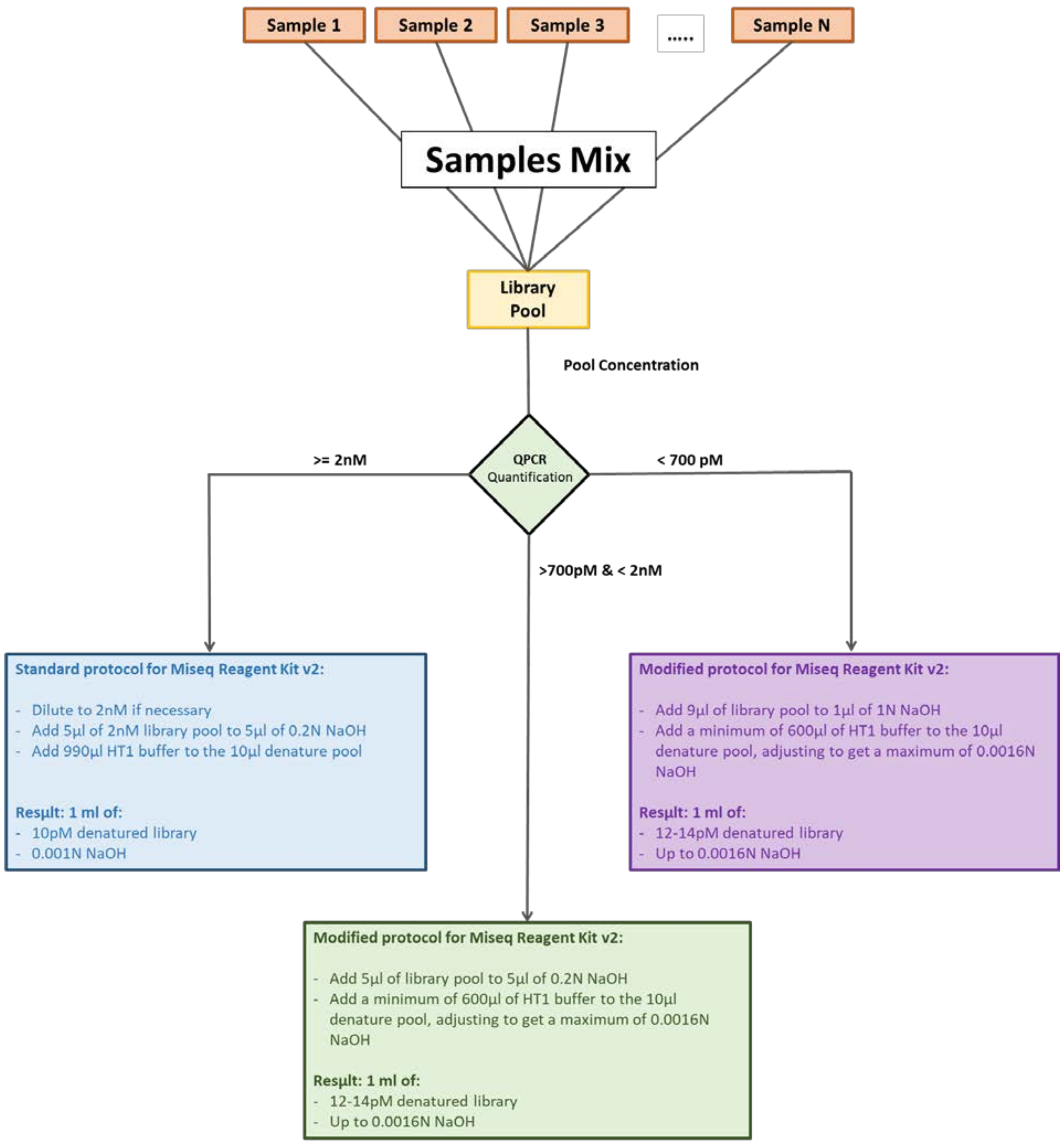

Figure 5: Decision tree for library sequencing based on QPCR results.

Mixing the samples:

This step is crucial for the success of the library sequencing. The objective is to get the closest equimolar mix of samples even though some of the samples may present a low concentration. For that reason we recommend to follow these steps:

1 Based on results from the QPCR, for each sample calculate the volume needed to reach equimolarity taking as reference the lowest concentration/s. If the difference between the lowest concentrations and the average concentrations is more than 4 times, use all volume for the lowest concentrations, then adjust rest of the samples to an equal concentration.

2 Draw a line at 20 µl approx.in a 1.5 ml Eppendorf.

3 Mix the calculated volumes of sample libraries (100 µl approx.) in the 1.5 µl Eppendorf.

4 Following manufacturer's instructions, concentrate the pool using a Savant SpeedVac Concentrator (Thermo Scientific) at room temperature until the volume reaches the 20 µl level.

5 Quantify the library pool as stated in "Step 4. Quantify each index-tagged library by QPCR."

Proceed to cluster amplification using the appropriate Illumina Paired-End Cluster Generation Kit, based on library pool quantification:

1 If pool concentration is higher or equal to 2nM:

- **a** Dilute to 2nM if necessary
- **b** Add 5µl of 2nM library pool to 5µl of 0.2N NaOH
- **c** Add 990µl HT1 buffer to the 10µl denature pool

2 If pool concentration is higher than 700pM but lower than 2nM:

- **a** Add 5ul of library pool to 5ul of 0.2N NaOH
- **b** Add a minimum of 600ul of HT1 buffer to the 10ul denature pool, adjusting to get around 0.0016N NaOH

3 If pool concentration is lower than 700pM:

- **a** Add 9ul of library pool to 1ul of 1N NaOH
- **b** Add a minimum of 600ul of HT1 buffer to the 10ul denature pool, adjusting to get around 0.0016N NaOH

CAUTION **Make sure NaOH concentration never exceeds 0.0025N.**

See Table 14 for kit configurations recommended for the sequencing of FFPE SureSelect^{QXT} pools. To do this step, refer to the manufacturer's instructions, using the modifications described in "Using the SureSelect^{QXT} Read Primers with Illumina's Paired-End Cluster Generation Kits" on next page.

Table 14: Illumina Kit Configuration Selection Guidelines for Miseq

Platform	Run Type	Read Length	SBS Kit Configuration	Chemistry
MiSeq	All Runs	2 × 150 bp	300 Cycle Kit	v2
MiSeq	All Runs	2 × 150 bp	300 Cycle Kit	v3

Using the SureSelect^{QXT} Read Primers with Illumina's Paired-End Cluster Generation Kits

To sequence the SureSelect^{QXT} libraries on Illumina's sequencing platforms, you need to use the following custom sequencing primers, provided in SureSelect QXT Library Prep Kit Box 2:

- SureSelect QXT Read Primer 1
- SureSelect QXT Read Primer 2
- SureSelect QXT Index Read Primer

These SureSelect^{QXT} custom sequencing primers are provided at 100 µM and must be diluted 1:200 in the corresponding Illumina primer solution, using the platform-specific instructions below:

For the MiSeq platform, combine the primers as shown in Table 16 on page 31.

NOTE It is important to combine the primers precisely in the indicated ratios. Be sure to use measured volumes of each solution; do not use volumes reported on vial labels when preparing the mixtures. Vortex each mixture vigorously to ensure homogeneity for proper detection of the indexes using the custom read primers.

Table 15: MiSeq platform **custom sequencing primer preparation**

Sequencing Read	Volume of SureSelect^{QXT} Primer	Volume of Illumina TruSeq Primer	Total Volume	Final Cartridge Position
Read 1	3 µl SureSelect QXT Read Primer 1 (brown cap)	597 µl HP10 (well 12)	0.6 ml	well 18
Index	3 µl SureSelect QXT Index Read Primer (clear cap)	597 µl HP12 (well 13)	0.6 ml	well 19
Read 2	3 µl SureSelect QXT Read Primer 2 (black cap)	597 µl HP11 (well 14)	0.6 ml	well 20

Nucleotide Sequences of SureSelect^{QXT} Dual Indexes

The nucleotide sequence of each SureSelect^{QXT} index is provided in the tables below.

Note that some index number assignments of the SureSelect^{QXT} P5 and P7 indexes differ from the index number assignments used by Illumina for indexes of similar or identical sequence.

Each index is 8 bases in length. Refer to Illumina's sequencing run setup instructions for sequencing libraries using 8-base indexes.

Table 16: SureSelect^{QXT} P7 Indexes 1 to 12

Index Number	Sequence
P7 Index 1 (P7 i1)	TAAGGCGA
P7 Index 2 (P7 i2)	CGTACTAG
P7 Index 3 (P7 i3)	AGGCAGAA
P7 Index 4 (P7 i4)	TCCTGAGC
P7 Index 5 (P7 i5)	GTAGAGGA
P7 Index 6 (P7 i6)	TAGGCATG
P7 Index 7 (P7 i7)	CTCTCTAC
P7 Index 8 (P7 i8)	CAGAGAGG
P7 Index 9 (P7 i9)	GCTACGCT
P7 Index 10 (P7 i10)	CGAGGCTG
P7 Index 11 (P7 i11)	AAGAGGCA
P7 Index 12 (P7 i12)	GGACTCCT

Table 17: SureSelect^{QXT} P5 Indexes 13 to 20

Index Number	Sequence
P5 Index 13 (P5 i13)	TAGATCGC
P5 Index 14 (P5 i14)	CTCTCTAT
P5 Index 15 (P5 i15)	TATCCTCT
P5 Index 16 (P5 i16)	AGAGTAGA
P5 Index 17 (P5 i17)	GTAAGGAG
P5 Index 18 (P5 i18)	ACTGCATA
P5 Index 19 (P5 i19)	AAGGAGTA
P5 Index 20 (P5 i20)	CTAAGCCT

Guidelines for Multiplexing with Dual-Indexed Samples

Agilent recommends following the dual index sample pooling guidelines shown in Table 18 for 16 reaction kits. These are designed to maintain colour balance at each cycle of the index reads on both ends. They also provide flexibility of demultiplexing as single or dual indexed samples in low-plexity experiments. One-base mismatches should also be allowed during demultiplexing in order to maximize sequencing output per sample.

Table 18: Dual index sample pooling guidelines for 16 Reaction Kits

Plexity of Sample Pool	Recommended SureSelect^{QXT} P7 Indexes	Recommended SureSelect^{QXT} P5 Indexes
1-plex	Any P7 index (i1 to i8)	Either P5 index (i13 or i14)
2-plex	P7 i1 and P7 i2 OR<br>P7 i2 and P7 i4	P5 i13 and P5 i14
3-plex	P7 i1, P7 i2 and P7 i4 OR<br>P7 i3, P7 i4 and P7 i6 OR<br>P7 i5, P7 i7 and P7 i8	P5 i13 and P5 i14 (as needed)
4- or 5-plex	P7 i1, P7 i2, P7 i4 and any additional P7 index(es) OR<br>P7 i3, P7 i4, P7 i6 and any additional P7 index(es) OR<br>P7 i5, P7 i7, P7 i8 and any additional P7 index(es)	P5 i13 and P5 i14 (as needed)
6- to 8-plex	Any combination of 6, 7, or 8 different P7 indexes	P5 i13 and P5 i14 (as needed)
9-to 16-plex	All eight P7 indexes (i1 to i8)	P5 i13 and P5 i14 (as needed)
