# Supplementary material for: Modified SureSelectQXT Target Enrichment Protocol for Illumina Multiplexed Sequencing of FFPE Samples
Source: Biol Proced Online. 2018 Oct 12;20:19. doi: 10.1186/s12575-018-0084-7 (PMC6182866; doi:10.1186/s12575-018-0084-7)
Supplement: Supplementary file 7 — Table S2. A) PTEN exon 1 primers for qcPCR. B) Preparation of quality control PCR Reaction mix. C) Thermal cycler program for Quality Control PCR. In blue the finally chosen number of cycles. (DOCX 18 kb) [file 12575_2018_84_MOESM7_ESM.docx]

Additional file 7: Table S2: A) PTEN exon 1 primers for qcPCR. B) Preparation of quality control PCR Reaction mix. C) Thermal cycler program for Quality Control PCR. In blue the finally chosen number of cycles.

**A**

| **Name** | **Orientation** | **Sequence** | **Annealing Tp** | **Insert Size** |
| --- | --- | --- | --- | --- |
| PTEN_EX1_F | Forward | 5´- GCAGCTTCTGCCATCTCTCT -3´ | 54ºC | 200 bp |
| PTEN_EX1_R | Reverse | 5´- CATCCGTCTACTCCCACGTT - 3´ |  |  |

B

| **Reagent** | **Volume for 1 reaction** |
| --- | --- |
| Nuclease-free water | 4.1 µl |
| MgCl 25 mM* | 1.5 µl |
| 10X PCR Buffer II* | 2.5 µl |
| dNTP mix 1,25mM** | 4 µl |
| 10 µlM PTEN_EX1_F | 1.25 µl |
| 10 µlM PTEN_EX1_R | 1.25 µl |
| AmpliTaq Gold DNA Polymerase (5 U/μL)* | 0.4 µl |
| **Total** | **15 µl** |

^*^ From the kit AmpliTaq Gold^TM^ Polymerase with Buffer II and MgCl_2_, Applied Biosystems^TM^, ref. N8080247.

^**^ Promega Corporation ref. U1240. To achieve required concentration, mix 12.5 µl from each of the 4 DNTPs at 100mM, then add 950 µl of nuclease free water.

**C**

| **Segment Number** | **Number of Cycles** | **Temperature** | **Time** |
| --- | --- | --- | --- |
| 1 | 1 | 94°C | 6 minutes |
| 2 | 25 | 94°C | 45 seconds |
|  |  | 54°C | 45 seconds |
|  |  | 72°C | 1 minute |
| 3 | 1 | 72°C | 7 minutes |
| 4 | 1 | 4°C | Hold |
